# Current Insights into Clinical, Molecular, and Therapeutic Approaches to Acute Respiratory Distress Syndrome

**DOI:** 10.3390/medsci14010134

**Published:** 2026-03-13

**Authors:** Manuel Gonzalez-Plascencia, Margarita L. Martinez-Fierro, Alfredo Salazar de Santiago, Ana G. Castañeda-Miranda, José I. Badillo-Almaraz, Idalia Garza-Veloz

**Affiliations:** 1 Molecular Medicine Laboratory, Academic Unit of Human Medicine and Health Sciences, Universidad Autónoma de Zacatecas, Carretera Zacatecas-Guadalajara Km.6, Ejido la Escondida, Zacatecas 98160, Mexico; manuelgonzalezcharro@gmail.com (M.G.-P.); jibadillo@hotmail.com (J.I.B.-A.); 2 Unidad Académica de Odontología, Universidad Autónoma de Zacatecas, Zacatecas 98160, Mexico; asalazar@uaz.edu.mx; 3 Laboratorio de Magnetismo Ambiental, Posgrado en Ingeniería para la Innovación Tecnológica, Unidad Académica de Ingeniería Eléctrica, Universidad Autónoma de Zacatecas, Zacatecas 98000, Mexico; agmiranda@uaz.edu.mx

**Keywords:** acute respiratory distress syndrome, biological heterogeneity, ARDS phenotypes, biomarkers, alveolar–capillary barrier, precision medicine

## Abstract

Acute respiratory distress syndrome (ARDS) remains a leading cause of morbidity and mortality in critically ill patients despite advances in supportive care and lung-protective ventilation. The syndrome is characterized by biological heterogeneity involving epithelial and endothelial injury, dysregulated inflammation, surfactant dysfunction, and impaired alveolar–capillary barrier integrity. This review integrates experimental, translational, and clinical evidence to examine the biological and molecular basis underlying ARDS, with particular emphasis on alveolar–capillary architecture, immune dysregulation, pulmonary mechanics, and the temporal evolution of diffuse alveolar damage. We further discuss emerging concepts in ARDS phenotyping and biomarker-based stratification as tools to address therapeutic heterogeneity and improve prognostic precision. Collectively, the evidence supports a shift from syndromic management toward biologically informed, precision-based approaches that may enable targeted interventions and improved clinical outcomes in ARDS.

## 1. Introduction

Acute respiratory distress syndrome (ARDS) is a severe form of acute hypoxemic respiratory failure characterized by diffuse inflammatory lung injury, increased alveolar–capillary permeability, and non-cardiogenic pulmonary edema [1,2,3]. Despite standardized definitions and advances in critical care, ARDS continues to impose a substantial global burden, with reported hospital mortality ranging from 30% to over 45% in severe cases, and even higher rates in resource-limited settings [1,2,3]. The COVID-19 pandemic further highlighted both the prevalence of ARDS and the limitations of current syndromic approaches to its management [2,4]. Clinically, ARDS is defined by acute onset, bilateral pulmonary infiltrates, and severe impairment of oxygenation not fully explained by cardiac failure [4,5]. However, previous evidence indicates that ARDS represents not a single disease entity, but a heterogeneous clinical syndrome encompassing distinct biological processes and injury patterns [6]. These include variable contributions of epithelial versus endothelial damage, differential activation of innate and adaptive immune pathways, surfactant dysfunction, coagulation abnormalities, and microvascular injury [7,8,9,10,11]. This biological heterogeneity has important implications for prognosis and treatment response [6,12]. Numerous pharmacologic interventions have failed to demonstrate benefit in unselected ARDS populations, suggesting that uniform therapeutic strategies may obscure meaningful effects within biologically distinct subgroups [12,13,14]. In this context, increasing attention has been directed toward mechanistic phenotyping, biomarker-driven stratification, and integration of molecular and physiological data to refine ARDS classification and guide therapy [12,15,16].

The aim of this narrative review is to synthesize current knowledge on the biological and molecular foundations of ARDS, linking structural lung injury, immune dysregulation, and pulmonary mechanics with emerging concepts in phenotyping and precision medicine.

## 2. Methods

This narrative review was based on a structured literature search conducted in PubMed, Scopus, and Web of Science. Although no strict date limits were applied, the review prioritized studies published within the last 5–7 years to capture recent advances, while incorporating seminal historical investigations essential for the mechanistic context.

Search terms included combinations of “acute respiratory distress syndrome,” “alveolar-capillary membrane,” “diffuse alveolar damage,” “pulmonary surfactant,” “endothelial injury,” “immune dysregulation,” “ARDS phenotypes,” and “biomarkers.” The reference list of relevant articles was also screened to identify additional key publications.

Inclusion criteria comprised peer-reviewed original experimental studies, translational investigations and clinical observational studies that directly contributed to the mechanistic understanding, phenotypic characterization, or therapeutic implications of ARDS. High-impact consensus statements and landmark reviews were included when they provided essential conceptual frameworks.

Exclusion criteria included non-peer-reviewed sources, editorials without primary data, case reports lacking mechanistic insight, and studies not directly relevant to ARDS pathophysiology or clinical interpretation. Given the narrative design, no formal PRISMA workflow or quantitative meta-analysis was performed. Instead, a qualitative synthesis was conducted to integrate structural, molecular, and clinical dimensions of ARDS.

## 3. Structural and Mechanistic Basis of ARDS

Understanding the biological and molecular basis of ARDS requires examining the alveolar–capillary membrane (ACM) as an integrated structural, mechanical, vascular, and immunological interface. Within this unit, epithelial–endothelial barrier integrity, surfactant-dependent surface tension regulation, extracellular matrix (ECM) mechanics, perfusion matching, and immune tolerance operate in coordinated balance to sustain gas exchange and fluid homeostasis. In ARDS, disruption of these coupled systems, barrier failure, surfactant inactivation, mechanical heterogeneity, ventilation–perfusion uncoupling, and immune amplification, drives the development of diffuse alveolar damage (DAD). The following sections analyze these interdependent mechanisms to provide a mechanistic framework for understanding the spatial heterogeneity, physiological instability, and dynamic progression characteristic of ARDS.

### 3.1. Barrier Disruption and Permeability Failure

**Figure 1:**
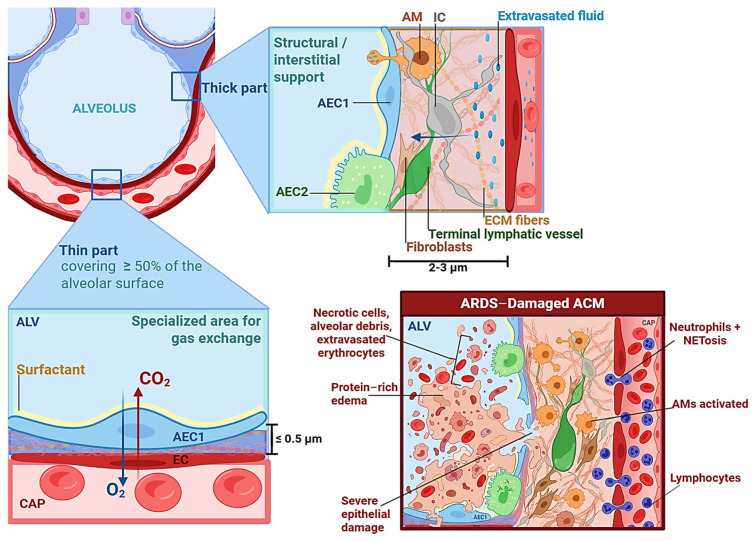
Structure and functional architecture of the alveolar–capillary membrane (ACM). The ACM consists of two major regions; the thin part (≤0.5 μm) covers most of the alveolar surface and is specialized for gas exchange. It includes alveolar epithelial type I cells (AEC1) adjacent to capillary endothelial cells (EC), often sharing a fused basement membrane to minimize diffusion distance. The surfactant layer, produced by alveolar epithelial type II cells (AEC2), lines the alveolar interface, reduces surface tension, and maintains alveolar stability. Oxygen (O_2_, blue arrows) diffuses from the alveolus (ALV) into the capillary (CAP), while carbon dioxide (CO_2_, red arrows) diffuses out. Thick part (2–3 μm): Provides structural and interstitial support. It contains AEC2, fibroblasts, interstitial cells (IC), extracellular matrix (ECM) fibers (collagen, elastin), alveolar macrophages (AMs), terminal lymphatic vessels, and capillary networks. This region supports tissue integrity, immune surveillance, and fluid/protein transport. In ARDS, the ACM is severely disrupted. The alveolar space contains necrotic cells, debris, and extravasated erythrocytes, with protein-rich edema and epithelial damage. On the capillary side, neutrophils undergoing NETosis, activated macrophages, and lymphocytes contribute to inflammation and barrier dysfunction, impairing gas exchange and promoting respiratory failure.

The ACM or air–blood barrier is the primary structural target of injury in ARDS [17,18]. It consists of a continuous alveolar epithelium, a continuous capillary endothelium, and a fused interstitium [19]. As illustrated in Figure 1, its organization into a thin side optimized for gas diffusion (≤0.5 µm) and a thick side supporting structural stability and lymphatic drainage enables efficient gas exchange but confers marked vulnerability to permeability failure during inflammatory injury [19,20]. Under physiological conditions, the thick side regulates limited fluid leakage and permits lymphatic clearance [20,21]. In ARDS, endothelial activation and junctional disruption overwhelm this compensatory capacity, leading to protein-rich alveolar edema and DAD [20,21]. 

The alveolar epithelium is composed predominantly of two cell types: (1) Alveolar epithelial type 1 cells (AEC1) cover approximately 90–95% of the alveolar surface area and are optimized for gas diffusion due to their extreme thinness and expansive cytoplasmic extensions [21,22]. (2) In contrast, alveolar epithelial type 2 cells (AEC2) occupy a smaller surface area but serve critical functions: surfactant synthesis and secretion, epithelial progenitor capacity for AEC1 regeneration, and active ion transport contributing to alveolar fluid clearance [23]. Thus, epithelial injury in ARDS compromises not only structural continuity but also surfactant homeostasis and regenerative potential [17,24].

Endothelial cells form the second critical barrier layer. Under physiological conditions, the pulmonary endothelium exhibits low hydraulic conductivity and a high reflection coefficient to plasma proteins, preserving oncotic gradients [20,22]. The pulmonary capillary endothelium regulates vascular tone, inflammation, and coagulation [8,25]. In ARDS, inflammatory signaling mediated by interleukin (IL)-1β, IL-6, and tumor necrosis factor alpha (TNF-α) activates nuclear factor kappa B (NF-κB) and signal transducer and activator of transcription 3 (STAT3) pathways, thereby promoting cytoskeletal contraction, junctional disassembly, and increased transendothelial permeability [8,26,27]. This endothelial dysfunction permits protein-rich plasma leakage into the interstitium and alveolar space, overwhelming lymphatic clearance and initiating permeability edema [22].

Alveolar macrophages (AMs) contribute to immune surveillance; however, in ARDS their pro-inflammatory polarization promotes cytokine release, endothelial activation, and further barrier destabilization [28]. ACM integrity depends on tight and adherens junctions, including claudins (claudin-3, -4, -18 in the epithelium; claudin-5 in the endothelium), occludin, VE-cadherin and Zonula Occludens-1 (ZO-1) [19,27,29]. Claudin-4 enhances barrier function, whereas claudin-3 promotes paracellular leak [29]. Inflammatory signaling in ARDS disrupts these junctional complexes, producing endothelial gap formation, protein extravasation, and permeability edema [27].

Finally, disruption of this delicate architecture in ARDS is reflected in measurable biomarkers: soluble receptor for advanced glycation end products (sRAGE) and surfactant proteins (SP-A/B/D) as indicators of epithelial injury; angiopoietin-2 (Ang-2) and von Willebrand factor (vWF) as markers of endothelial activation; and Club cell secretory protein 16 (CC16) leakage as a marker of club cell integrity [30,31,32,33].

### 3.2. Surfactant Dysfunction and Alveolar Instability

Pulmonary surfactant, produced by AEC2 cells, is a lipoprotein complex composed of approximately 90% lipids and 10% proteins [9,34]. An essential function of pulmonary surfactant is to dynamically regulate surface tension at the alveolar air–liquid interface, preventing end-expiratory collapse and minimizing the work of breathing [9,20,21,35]. Surfactant operates as a biophysically adaptive film that modulates tension according to alveolar surface area, becoming densely packed and nearly solid at end-expiration to provide maximum resistance against collapse, and re-expanding into a more fluid state during inspiration to facilitate reopening [9].

Alveolar stability requires very low surface tension approaching near-zero values, achieved through a dipalmitoyl-phosphatidylcholine (DPPC)-enriched film that, upon compression, reduces tension to 1–2 mN/m [34,35,36]. During inspiration, the film re-expands and becomes more fluid, allowing controlled alveolar reopening [21,35]. Loss of this dynamic compressibility, such as with SP-B inactivation or DPPC dilution observed in ARDS, predisposes to atelectasis and regional instability [20,34,36].

Under physiological conditions, extravascular lung water is tightly regulated through balanced hydrostatic and oncotic forces and an efficient lymphatic clearance [20,35,37]. This equilibrium depends not only on endothelial integrity (discussed above) but also on intact surfactant function. In ARDS, increased permeability permits plasma proteins to enter the alveolar space, where they directly inactivate surfactant [9,24,35,38].

Importantly, surfactant dysfunction is not merely a consequence of edema but a mechanistic amplifier of fluid accumulation [35,39]. Elevated surface tension increases alveolar elastic recoil pressure, generating greater traction forces on the interstitium and enhancing the transvascular filtration gradient [9,20,35,36]. Two complementary mechanisms explain this amplification: (1) a transepithelial effect, where elevated surface tension promotes alveolar collapse and increases recoil pressure, lowering interstitial pressure and favoring microvascular filtration; and (2) a transendothelial effect, where computational models based on the revised Starling principle show that high surface tension reduces interstitial pressure enough to overcome the counteracting effect of positive end-expiratory pressure (PEEP), thereby promoting fluid flux into the alveoli [9,20,35].

Thus, surfactant inactivation and permeability edema act synergistically in ARDS. Initial epithelial–endothelial injury permits protein leakage; protein-rich edema fluid impairs surfactant function [24,35]; increased surface tension promotes collapse and further filtration; and progressive alveolar flooding perpetuates instability [35,39]. This establishes a vicious cycle: initial injury inactivates surfactant, elevated tension promotes edema, edema further inactivates surfactant, culminating in rapid and diffuse alveolar collapse [9,35]. This feed-forward loop constitutes a central mechanistic driver of diffuse alveolar collapse in ARDS [9,35].

### 3.3. Mechanical Heterogeneity and Ventilator-Induced Lung Injury Susceptibility

The mechanical stability of the lung parenchyma depends on the interaction between the ECM (elastin, collagen, proteoglycans) and the surfactant system [9,21,24]. Together, these elements determine alveolar micromechanics and global lung elastance [21,40,41]. In ARDS, this integrated mechanical network becomes profoundly disrupted [39,42].

Compliance reflects the distensibility of the respiratory system, whereas elastance represents its stiffness [43]. The hallmark mechanical abnormality in ARDS is a marked reduction in lung compliance, primarily driven by edema, inflammation, surfactant dysfunction, and alveolar collapse [35,39]. In pulmonary ARDS, chest wall compliance (CCW) may remain normal, whereas in extrapulmonary ARDS, such as in obesity or ascites, CCW can also be reduced [14]. Differentiating lung from chest wall mechanics using transpulmonary pressure, derived from esophageal pressure as a surrogate of pleural pressure, is essential to optimize protective ventilation strategies [14,44].

Beyond global compliance reduction, ARDS is characterized by pronounced spatial heterogeneity [40]. Alveoli are interconnected within a three-dimensional extracellular matrix, and under normal conditions alveolar interdependence provides structural stabilization through tethering forces [21]. This alveolar interdependence acts as a secondary stabilizing mechanism complementary to surfactant. In ARDS, disruption of this structural interdependence produces a heterogeneous mosaic of atelectatic, edematous, and overdistended regions [21].

Under mechanical ventilation, such heterogeneity produces localized stress amplification at the interfaces between aerated and non-aerated units [39,42]. These “stress raisers” predispose to ventilator-induced lung injury (VILI) through cyclic recruitment–derecruitment (atelectrauma) and regional overdistension (volutrauma) [9,21]. Thus, mechanical injury in ARDS is not solely a function of airway pressure magnitude but of uneven stress distribution within a structurally heterogeneous lung [21,39].

Airway resistance plays a comparatively minor role in ARDS pathophysiology [14,45]. Although resistance in distal airways follows Poiseuille principles and is influenced by airway radius [46], ARDS is primarily a compliance-driven, not resistance-driven, disorder [14]. Clinically, this distinction delineates ARDS from obstructive diseases such as asthma or chronic obstructive pulmonary disease, where airflow limitation predominates. Clinically, this distinction delineates ARDS from obstructive diseases such as asthma or chronic obstructive pulmonary disease COPD, where airflow limitation predominates. In ARDS, the defining mechanical abnormality is reduced compliance with only modest changes in airway resistance [39,47]. Therefore, ARDS represents a mechanically heterogeneous system in which reduced compliance, disrupted interdependence, and regional stress concentration collectively increase susceptibility to VILI during ventilatory support [39,41,42].

### 3.4. VA/Q Mismatch and Vascular Dysregulation

Efficient pulmonary gas exchange requires tight matching between alveolar ventilation (V) and perfusion (Q), regulated by gravitational perfusion gradients and active mechanisms such as hypoxic pulmonary vasoconstriction (HPV) [48,49]. In ARDS, this coupling collapses at multiple levels (ventilatory, vascular and microthrombotic) producing simultaneous shunt and dead space amplification [50,51,52,53].

Physiological dead space (Vdphys) represents wasted ventilation in units with high or infinite VA/Q ratios and is quantified using the Bohr equation [50,54]. It includes anatomical dead space and alveolar dead space (Vdalv), the latter reflecting ventilated but poorly perfused alveoli [54,55]. In ARDS, Vdalv rises markedly due to microvascular thrombosis, endothelial injury, vascular obliteration, and capillary compression from overdistension [51,52,56]. A Vd/Vt ratio exceeding 0.35–0.40 indicates severe ventilatory inefficiency and correlates with increased intensive care unit (ICU) mortality [57]. Accordingly, volumetric capnography-derived dead space monitoring provides prognostic and ventilatory guidance value [57].

**Figure 2:**
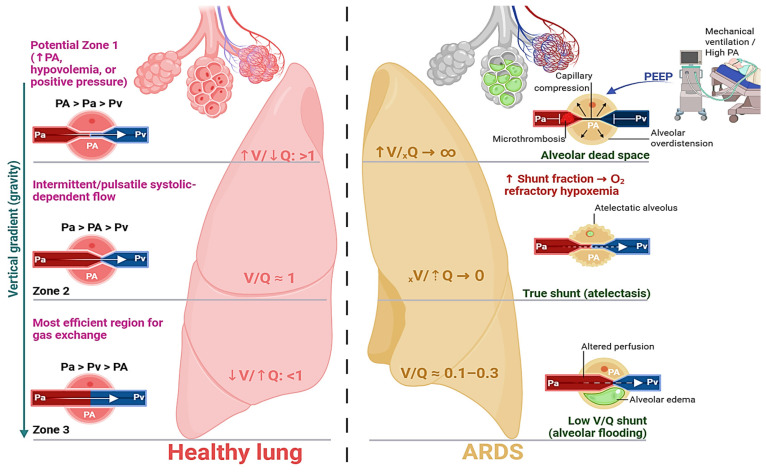
West’s lung zones and alterations in ventilation–perfusion (V/Q) relationships in ARDS. In the healthy lung (**left**), gravitational gradients determine perfusion and ventilation distribution across West’s physiological zones. Zone 1 (PA > Pa > Pv) exhibits intermittent or absent flow (V/Q > 1), Zone 2 (Pa > PA > Pv) maintains balanced V/Q ≈ 1, and Zone 3 (Pa > Pv > PA) favors efficient gas exchange with slightly lower V/Q < 1. In ARDS (**right**), these regional relationships are disrupted. Alveolar dead space (V/Q → ∞) results from capillary compression, microthrombosis, or high PEEP, leading to ventilation without perfusion. Shunt I (V/Q ≈ 0) occur in atelectatic or collapsed alveoli that are perfused but not ventilated. Alveolar flooding and edema-related V/Q mismatch show partial ventilation–perfusion imbalance (V/Q ≈ 0.1–0.3) caused by alveolar fluid accumulation and altered perfusion. Together, these abnormalities generate severe hypoxemia and impaired gas exchange typical of ARDS.

Perfusion distribution follows gravitational gradients but is dynamically influenced by cardiac output and intrathoracic pressure [49,54,58,59]. The West zones model conceptualizes this distribution according to the relationship between alveolar pressure (PA), arterial pressure (Pa), and venous pressure (Pv) [54]. In ARDS, mechanical ventilation—particularly PEEP—profoundly alters these relationships [49,60]. By increasing alveolar pressure, PEEP can convert Zone 3 regions into Zone 2 and Zone 2 into Zone 1, thereby generating ventilated but underperfused territories (functional dead space) [50,54]. This duality underscores the narrow therapeutic window of PEEP: insufficient levels perpetuate shunt (VA/Q = 0), whereas excessive levels increase alveolar dead space and reduce compliance [54,57,61,62]. Figure 2 integrates West’s zonal physiology with ARDS-related VA/Q disruption, illustrating how recruitment and overdistension coexist within the same lung, simultaneously reducing shunt while expanding dead space. 

HPV constitutes the principal active mechanism preserving VA/Q matching [48,63]. Triggered by reduced alveolar PO_2_, it induces precapillary vasoconstriction, diverting flow from hypoventilated regions toward better-ventilated units [48,49,63]. In ARDS, endothelial injury and inflammatory mediator release (including NO and prostacyclin) blunt or abolish HPV [20,48,52,54,56]. Consequently, perfusion persists in non-ventilated or collapsed alveoli, amplifying intrapulmonary shunt [49,55]. Refractory hypoxemia in ARDS therefore reflects combined vascular dysregulation and structural heterogeneity: extensive VA/Q = 0 regions from alveolar collapse, failure of compensatory HPV, and superimposed microthrombotic perfusion defects that elevate dead space [36,49,50,54,57]. Computational modeling confirms that loss of regional coupling profoundly reduces effective oxygen uptake and homogenization of capillary oxygenation [64,65,66].

Thus, ARDS is characterized not by isolated shunt or dead space, but by their coexistence, driven by mechanical ventilation, vascular injury, and failed hypoxic vasoregulation, creating a physiologically unstable system highly sensitive to ventilatory and hemodynamic perturbations [50,67,68,69].

### 3.5. Immune Amplification and Loss of Tolerance

The pulmonary immune system operates under a tightly regulated tolerogenic framework that preserves gas exchange while preventing excessive inflammation [70,71]. Under homeostatic conditions, AMs and dendritic cells (DCs) sustain tolerance: AMs exhibit an M2-like phenotype, releasing IL-10 and TGF-β to promote FoxP3^+^ regulatory T cells (Tregs), while DCs favor Treg polarization and IL-10 secretion [28,71,72,73,74].

ARDS represents a collapse of this tolerogenic equilibrium [75,76]. Danger signals, suchs as pathogen-associated molecular patterns (PAMPs) and damage-associated molecular patterns (DAMPs), released from injured epithelial and endothelial cells engage pattern recognition receptors (PRRs, for example toll-like receptors, TLRs) on AMs and alveolar epithelial cells, triggering innate immune activation [71,75,76]. AMs shift toward a pro-inflammatory M1 phenotype, releasing IFN-α/β, IL-6, TNF-α, IL-12, and chemokines such as IL-8, which recruit neutrophils [28,71,77,78]. Subsequent amplification via IL-1β, TNF-α, IFN-γ, reactive oxygen species (ROS), and inducible nitric oxide (NO) activates NF-κB and STAT3 pathways, destabilizing epithelial and endothelial junctions within the ACM and increasing permeability [79,80].

As ARDS progresses, neutrophils become the dominant effector population [81,82]. Tissue injury is largely host-mediated through NETosis, whereby neutrophils release extracellular traps composed of DNA, histones, neutrophil elastase (NE), and myeloperoxidase (MPO) [71,82]. These components directly damage alveolar epithelium and capillary endothelium: histones disrupt plasma membranes and induce calcium-dependent cell death, while NE degrades endothelial cadherins, compromising junctional integrity and accelerating microvascular injury [81,83,84]. Thus, immune amplification directly feeds structural barrier failure [84].

DCs, normally tolerogenic, become activated in the inflammatory milieu. HMGB1 enhances DC maturation via PI3K/Akt/mTOR signaling, promoting pro-inflammatory cytokine release [71,74,75,85,86,87]. Although DCs and Tregs can exert protective effects for example in transfusion-related acute lung injury (TRALI), via IL-10, this regulatory axis is functionally overwhelmed in ARDS [88,89]. Innate lymphoid cells (particularly ILC2s) and tissue-resident memory T cells (TRMs) contribute to epithelial repair and barrier immunity through amphiregulin production and rapid antigen-specific responses [90,91]. However, their reparative functions are insufficient to counterbalance the magnitude of inflammatory injury during severe ARDS [92,93]. Circulating monocytes further amplify inflammation, and alterations in the lung microbiota may disrupt immune tolerance signaling, although their precise contribution in ARDS remains incompletely defined [72,94,95,96].

SP-A and SP-D constitute an additional immunoregulatory layer [97]. These collectins act as opsonins, enhance pathogen clearance, and modulate macrophage activation while limiting excessive cytokine production [97,98,99,100]. They suppress inflammatory signaling in the absence of pathogens (e.g., via SIRPα interaction) and inhibit TNF-α production in myeloid cells [97,99]. In ARDS, barrier disruption permits leakage of SP-D into circulation, where elevated serum levels correlate with disease severity and reflect alveolar damage [31,71,101]. Loss of compartmentalized surfactant collectin function therefore contributes to unchecked inflammation and impaired resolution [24,35,102].

Collectively, ARDS reflects a transition from regulated immune tolerance to uncontrolled inflammatory amplification, in which macrophage reprogramming, neutrophil-mediated cytotoxicity, DCs activation, and loss of surfactant immunomodulation converge to destabilize the ACM [24,82,85,102]. The principal cellular and molecular networks governing pulmonary tolerance and inflammatory amplification—including regulatory macrophages, tolerogenic DCs, Tregs, innate lymphoid cells, and inflammatory effector populations—are summarized in Supplementary Table S1. In addition, the following references supporting these mechanisms have been incorporated into the manuscript: [103,104,105,106,107,108,109,110,111,112].

## 4. ARDS: Etiology, Risk Factors and Clinical Approach

### 4.1. Etiological Spectrum of ARDS

From a pathophysiological standpoint, ARDS is broadly classified into direct (pulmonary) and indirect (extrapulmonary) forms according to the primary site of injury. Direct ARDS arises from insults within the lung parenchyma that primarily damage the ACM, whereas indirect ARDS results from systemic inflammatory conditions such as sepsis, pancreatitis, or major trauma that secondarily target the pulmonary endothelium [113]. Despite differing triggers, ACM disruption constitutes the unifying pathological event, leading to increased permeability, non-cardiogenic pulmonary edema, and inflammatory activation that culminate in respiratory failure [1].

#### 4.1.1. Epithelial-Dominant Injury (Direct Pulmonary Etiologies)

Pneumonia of bacterial, viral, or fungal origin represents the most frequent cause of ARDS [1]. Pathogen invasion of distal airspaces activates alveolar macrophages and promotes neutrophil recruitment [81]. Dysregulated activation results in the release of IL-1β, IL-6, IL-8, and TNF-α, together with proteases and ROS, leading to epithelial apoptosis and necrosis, disruption of intercellular junctions, and increased epithelial permeability [81,114]. Loss of epithelial barrier integrity permits leakage of protein-rich fluid into the alveolar space, a defining feature of DAD [81,114].

COVID-19–associated ARDS demonstrates features of direct epithelial infection with secondary systemic amplification [115]. SARS-CoV-2 infects alveolar epithelial cells via ACE2, producing viral pneumonitis and cytopathic injury [115]. Severe cases are associated with exaggerated cytokine release and endothelial activation, resembling inflammatory patterns observed in extrapulmonary ARDS [115].

Aspiration of gastric contents induces acute epithelial injury through low-pH-mediated membrane denaturation and necrosis, followed by secondary cytokine-driven neutrophilic inflammation [1,116]. This sequence accelerates barrier disruption and promotes alveolar flooding [1,116].

Pulmonary contusion, inhalation injury, and drowning also produce predominant epithelial disruption. Pulmonary contusion damages alveolar epithelium and microvascular endothelium, increasing permeability and promoting intra-alveolar hemorrhage and edema [81,113]. Inhalation injury causes thermal and chemical necrosis of the airway and alveolar epithelium [116]. Drowning results in surfactant washout, alveolar instability, and inflammation [116].

E-cigarette or vaping-associated lung injury (EVALI) has been associated with progression to ARDS [117,118]. Vitamin E acetate disrupts surfactant function and accumulates in alveolar macrophages, contributing to lipotoxic stress and inflammatory activation [118]. Thermal degradation of Tetrahydrocannabinol generates toxic aldehydes that act as pulmonary irritants [118]. Suppression of WW domain-containing oxidoreductase (*WWOX*) has been linked to increased alveolar permeability and neutrophilic infiltration in experimental models [117].

**Figure 3:**
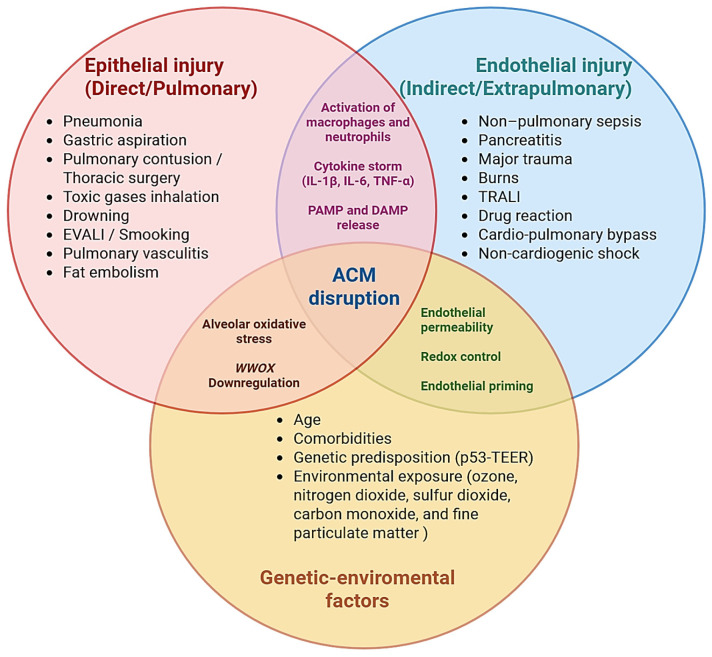
Integrated etiological domains and convergent mechanisms in ARDS. The diagram depicts the principal pathogenic domains of ARDS, highlighting the overlap between epithelial injury (direct/pulmonary), endothelial injury (indirect/extrapulmonary), and genetic–environmental modulators. Pulmonary insults (e.g., pneumonia, gastric aspiration, thoracic trauma, EVALI) primarily affect the alveolar epithelium, whereas extrapulmonary causes (e.g., sepsis, pancreatitis, TRALI, drug-induced toxicity) predominantly target the vascular endothelium. Both pathways converge through macrophage/neutrophil activation, PAMP/DAMP release, and a cytokine storm (IL-1β, IL-6, TNF-α), culminating in disruption of the alveolo-capillary membrane (ACM). Genetic and environmental factors, including p53-TEER polymorphisms, *WWOX* downregulation, oxidative stress, and chronic pollutant exposure (ozone, nitrogen dioxide, sulfur dioxide, carbon monoxide, fine particulate matter), further modulate susceptibility. The central overlap underscores ACM disruption as the final common pathway integrating inflammatory, oxidative, and structural mechanisms, thereby defining ARDS heterogeneity and informing potential targeted therapies.

Figure 3 schematically illustrates these epithelial-dominant mechanisms, depicting primary AEC injury, surfactant dysfunction, intra-alveolar cytokine amplification, and subsequent protein-rich edema formation within the alveolar space in direct ARDS [1,81,113,114]. 

#### 4.1.2. Endothelial-Dominant Injury (Indirect or Extrapulmonary Etiologies)

Sepsis of non-pulmonary origin, including peritonitis, pancreatitis, and urosepsis, remains the most frequent precipitant of extrapulmonary ARDS (ARDSexp) [1,3]. Circulating PAMPs and DAMPs initiate a systemic inflammatory response characterized by high concentrations of TNF-α, IL-1β, and other mediators that secondarily target the pulmonary vasculature [119]. Because the lung receives the entire cardiac output, the pulmonary capillary network is continuously exposed to these circulating factors, resulting in endothelial activation, disruption of intercellular junctions, increased vascular permeability, and leukocyte transmigration into the interstitium and alveolar space [114].

Major trauma and extensive burns induce a sterile systemic inflammatory response driven by DAMP release, activating signaling pathways similar to those observed in sepsis and culminating in pulmonary endothelial injury [81,119]. TRALI represents a prototypical iatrogenic model of endothelial-dominant ARDS [120]. Donor-derived anti-HLA or anti-HNA antibodies react with recipient neutrophils, leading to their activation and sequestration within the pulmonary microcirculation. Activated neutrophils release proteases and ROS that injure the capillary endothelium, resulting in abrupt capillary leak and non-cardiogenic pulmonary edema [120].

Drug-induced lung injury constitutes another extrapulmonary pathway to ARDS. Several chemotherapeutic agents promote endothelial dysfunction through oxidative and inflammatory mechanisms. Bleomycin and mitomycin-C enhance ROS generation; gemcitabine has been associated with cytokine-mediated capillary leak; cytarabine exerts direct parenchymal toxicity; and vinca alkaloids such as Vincristine disrupt endothelial microtubule architecture [121]. Non-oncologic agents, including Amiodarone and Nitrofurantoin, are also recognized triggers [121]. Amplification through the GM-CSF pathway increases neutrophil adhesion to activated endothelium, further exacerbating vascular permeability [121].

#### 4.1.3. Genetic and Environmental Modulators of Susceptibility

Host-related factors significantly influence susceptibility to ARDS. Advanced age, chronic comorbidities, and immune or oncologic disorders increase vulnerability through baseline systemic inflammation, endothelial fragility, and impaired repair capacity [122,123]. Genetic predisposition has been associated with polymorphisms and regulatory variants affecting pathways involved in cell proliferation, apoptosis, and redox homeostasis [122,123]. Among these, the p53 signaling axis has emerged as a regulator of endothelial barrier integrity. Experimental inhibition of p53 increases ROS generation, reduces transendothelial electrical resistance (TEER), and promotes pulmonary vascular dysfunction [124,125].

Environmental exposure further modulates risk. Chronic inhalation of pollutants—including ozone (O_3_), nitrogen dioxide (NO_2_), sulfur dioxide (SO_2_), carbon monoxide (CO), and fine particulate matter (PM2.5)—has been independently associated with increased ARDS incidence, even at concentrations considered acceptable under current regulatory thresholds [126,127]. These agents induce oxidative stress, epithelial dysfunction, and low-grade inflammatory activation, establishing a pulmonary “priming” state that amplifies injury following secondary insults such as sepsis, trauma, or aspiration [126]. Ozone exposure, in particular, enhances oxidative epithelial damage and increases ACM permeability, with amplified effects observed in smokers and trauma patients [126,127].

### 4.2. Clinical Presentation

ARDS manifests with acute onset dyspnea, tachypnea, and hypoxemia refractory to supplemental oxygen within one week of a known clinical insult [113,128]. Decreased lung compliance secondary to inflammatory edema increases the work of breathing and may culminate in respiratory muscle fatigue and ventilatory failure [3].

Hypoxemia and impaired ventilation can produce neurological alterations, including confusion or agitation, attributable to arterial desaturation, hypercapnia, or systemic hypoperfusion [128]. Concurrent systemic release of IL-1β, TNF-α, and IL-6 contributes to early multiple organ dysfunction syndrome, supporting the concept of ARDS as a systemic inflammatory condition [22]. Tachycardia is frequently observed, and central cyanosis reflects severe arterial hypoxemia [128]. The defining physiological abnormality is refractory hypoxemia caused by intrapulmonary shunt, in which perfusion of non-ventilated or fluid-filled alveoli prevents effective oxygenation across the ACM [3,113,114]. This mechanism underlies the clinical rationale for PEEP to recruit alveoli and reduce shunt fraction rather than relying exclusively on increased FiO_2_ [1]. Diffuse crackles may be present but are nonspecific. Diagnostic criteria require exclusion of hydrostatic (cardiogenic) pulmonary edema under the Berlin definition [113,128].

### 4.3. Diagnostic Criteria

The definition of ARDS has undergone refinement beyond the 2012 Berlin consensus in response to limitations identified during the COVID-19 pandemic and the widespread use of noninvasive respiratory support [2,5]. The updated framework revises four core domains: timing, imaging, origin of edema, and oxygenation.

1. **Timing.** ARDS requires acute onset, defined as new or worsening respiratory symptoms within one week of a known clinical insult [2]. This temporal boundary differentiates ARDS from subacute or chronic interstitial and inflammatory lung diseases that may also present with bilateral infiltrates, including idiopathic pulmonary fibrosis exacerbations, organizing pneumonia, acute eosinophilic pneumonia, or malignancy-related processes [1,128].
2. **Imaging.** Bilateral pulmonary opacities remain mandatory. However, lung ultrasound is now accepted as an alternative to chest radiography or computed tomography. Bilateral B-lines and/or consolidations fulfill the imaging criterion, expanding diagnostic applicability in resource-limited or critical care settings where CT is unavailable [2].
3. **Origin of edema.** Respiratory failure must not be fully explained by cardiac failure or fluid overload. When risk factors are absent or uncertainty persists, objective cardiac assessment, preferably echocardiography, is recommended to exclude hydrostatic pulmonary edema [1,2].
4. **Oxygenation.** The most substantive modification concerns respiratory support and gas exchange thresholds. ARDS can now be diagnosed in nonintubated patients receiving high-flow nasal oxygen (HFNO ≥ 30 L/min) or CPAP/NIV delivering ≥ 5 cmH_2_O of PEEP [2]. The PaO_2_/FiO_2_ (P/F) ratio thresholds are retained for severity stratification, and a SpO_2_/FiO_2_ (S/F) ratio ≤ 315 is validated as an alternative when arterial blood gases are unavailable, provided SpO_2_ ≤ 97% to ensure reliability along the oxyhemoglobin dissociation curve [2]. The New Global Definition formally recognizes “nonintubated ARDS,” applying identical oxygenation cutoffs for severity classification.

Comparative elements between the Berlin and New Global Definitions are summarized in Table 1.

In resource-limited settings, the updated definition removes strict requirements for minimum PEEP levels, specific devices, or detailed ventilatory parameters, enabling diagnosis based on clinical context, imaging, and oxygenation metrics adapted to available infrastructure [2].

## 5. Phases of ARDS Progression

### 5.1. Phases of ARDS and Diffuse Alveolar Damage

**Figure 4:**
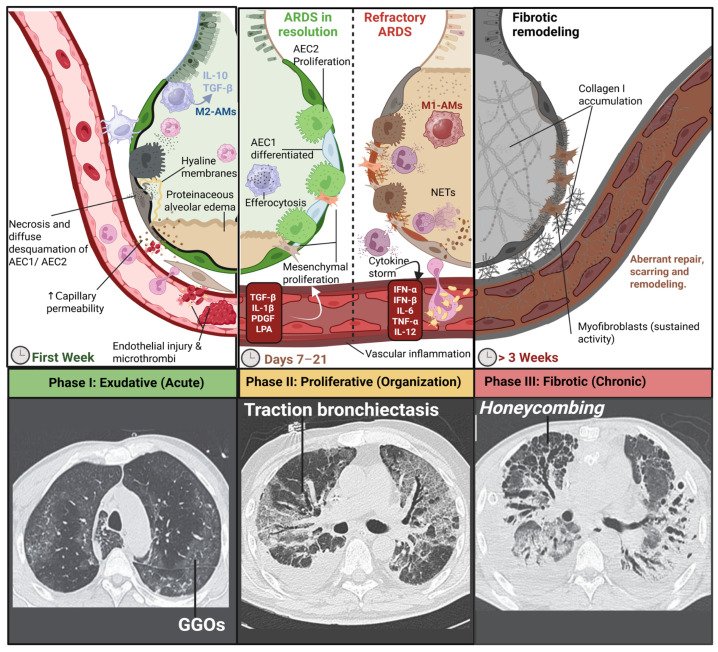
Sequential pathophysiological and radiological evolution of Acute Respiratory Distress Syndrome (ARDS). The image illustrates the progression of ARDS through three phases: Exudative (days 1–7), Proliferative (days 7–21), and Fibrotic (>21 days). Key cellular events, inflammatory mediators, and repair mechanisms are highlighted. Early injury to alveolar epithelial cells type I and II (AEC1/AEC2) and pulmonary endothelium leads to proteinaceous edema and hyaline membrane formation. Subsequent resolution may occur via AEC2 proliferation and differentiation into AEC1, or progress toward persistent inflammation characterized by a cytokine storm (e.g., TGF-β, IL-1β, PDGF, LPA, IFN-α/β, IL-6, TNF-α, IL-12). Chronic evolution culminates in fibrosing alveolitis, sustained myofibroblast activity, and collagen type I (Col-I) deposition. Radiological correlates include ground-glass opacities (GGOs), crazy paving, and honeycombing patterns on computed tomography (CT), reflecting the underlying histopathological changes. The chest computed tomography images are included for illustrative purposes only, do not correspond to patients included in the present review, and were adapted from Zompatori et al., Eur Respir Rev (2014), licensed under CC BY-NC 4.0 [131].

Diffuse alveolar damage constitutes the classic histopathological correlate of ARDS and is traditionally regarded as its defining morphological substrate [129]. The pathological evolution of ARDS is described as a temporal progression through three partially overlapping phases: an exudative (acute) phase, a proliferative or organizing (subacute) phase, and a fibrotic (chronic) phase [113,130]. The transition across these stages reflects the dynamic structural remodeling of the ACM and underpins the natural history of lung injury in ARDS [129]. Figure 4 synthesizes this progression, integrating the characteristic histopathological findings of each phase with their corresponding radiological correlates across the disease continuum.

Importantly, histopathological confirmation of DAD is not universal among patients fulfilling clinical ARDS criteria. Biopsy and autopsy series consistently demonstrate that only a subset of clinically defined ARDS cases exhibit DAD on examination [132]. The reported prevalence of DAD in clinical ARDS is approximately 50%, ranging between 45% and 65% [133]. Moreover, DAD prevalence correlates with oxygenation severity according to the Berlin criteria [2,133]. In a prospective autopsy study, DAD was identified in 12% of mild ARDS cases, 40% of moderate cases, and 58% of severe cases [133,134].

Contemporary longitudinal imaging data further substantiate the clinical relevance of this staged model of DAD. In a prospective cohort of mechanically ventilated COVID-19 ARDS survivors, Stoian et al. [135] reported fibrotic-like abnormalities in 87.5% of patients at 6-month follow-up, predominantly mild-to-moderate in severity, characterized by persistent ground-glass opacities (GGOs), reticulation, traction bronchiectasis, and, in a subset, honeycombing. Importantly, the extent of fibrosis was not significantly associated with the duration or modality of mechanical ventilation, underscoring the multifactorial nature of fibroproliferative remodeling beyond ventilator-induced lung injury alone. These findings reinforce the concept that, even in the absence of histopathological confirmation, the temporal evolution from acute alveolar injury to organizing and fibrotic remodeling is frequently mirrored in the longitudinal radiologic trajectory of ARDS survivors.

#### 5.1.1. Acute (Exudative) Phase

The exudative phase constitutes the initial histopathological expression of DAD, developing within hours and predominating during the first week after injury [132,136] (Figure 4). It is characterized by increased capillary permeability with protein-rich alveolar edema secondary to endothelial and epithelial barrier failure [113]. Plasma proteins, erythrocytes, and inflammatory cells accumulate within the interstitium and alveolar spaces, resulting in alveolar flooding [113]. Concomitant necrosis and desquamation of AEC1 expose the basement membrane and favor deposition of hyaline membranes composed of fibrin, cellular debris, and altered surfactant, a defining lesion of early DAD [129]. Endothelial activation further promotes coagulation cascade activation and formation of fibrin–platelet microthrombi within pulmonary capillaries, contributing to perfusion abnormalities and pulmonary hypertension [113,137]. Clinically, this phase corresponds to acute hypoxemic respiratory failure with markedly reduced compliance and increased physiological dead space [113]. Surfactant inactivation and AEC2 injury exacerbate alveolar instability and shunt physiology [137]. Radiographically, chest imaging evolves from subtle or normal findings in the first 24–48 h to bilateral alveolar opacities and consolidations [131]. Computed tomography (CT) demonstrates GGOs reflecting partial alveolar filling and consolidation representing complete airspace occupation, typically with dependent predominance [131,138].

#### 5.1.2. Proliferative (Organizing) Phase

The proliferative phase emerges between days 2 and 7 and predominates during the second to third weeks [129,132]. It reflects the attempt to clear intra-alveolar exudate and restore epithelial integrity. Surviving AEC2 proliferate and migrate along the denuded basement membrane, subsequently differentiating into AEC1 to re-establish the gas-exchange surface [113,129,136]. In parallel, alveolar macrophages remove debris and residual hyaline material [137]. Simultaneously, fibroblasts and myofibroblasts migrate and deposit provisional extracellular matrix composed primarily of fibronectin and type III collagen under the influence of mediators such as TGF-β and PDGF [139,140,141,142]. Histologically, this phase is defined by epithelial hyperplasia and interstitial or intra-alveolar fibroproliferation [136,140].

Clinical evolution depends on the balance between re-epithelialization and fibroproliferation. Effective epithelial repair is associated with improving oxygenation and compliance, whereas persistent fibroproliferation leads to prolonged ventilator dependence and non-resolving ARDS [113,141,142,143,144]. CT imaging demonstrates transition from GGOs and consolidation toward reticulation and traction bronchiectasis, reflecting organizing fibrosis; these findings are associated with worse prognosis when extensive [131,138,145].

#### 5.1.3. Chronic (Fibrotic) Phase

The fibrotic phase develops in a subset of patients in whom repair fails and fibroproliferation becomes dominant, typically after 2–3 weeks [129,143,146,147]. Acute neutrophilic inflammation subsides, but persistent myofibroblast activity drives replacement of provisional matrix by dense collagen type I deposition within interstitium and former alveolar space [142,147]. This results in irreversible architectural distortion, obliteration of alveolar–capillary units, and cystic remodeling lined by bronchiolar epithelium [142,147].

Clinically, advanced fibrosis manifests as refractory ventilator dependence, severe ventilation–perfusion mismatch, increased physiological dead space, and secondary pulmonary hypertension due to vascular remodeling [137,143]. CT imaging shows dominant coarse reticulation, extensive traction bronchiectasis, and honeycombing, representing end-stage architectural destruction [131,138,145] (Figure 4).

## 6. Advances Towards Precision Medicine: The Role of Phenotyping and Biomarkers in ARDS

### 6.1. Subphenotypes of ARDS

Although ARDS severity is clinically classified as mild, moderate, or severe according to standard criteria [5], its marked biological heterogeneity has led to the identification of subphenotypes and endotypes that may better explain differences in prognosis and treatment response [15,148]. A phenotype refers to the observable and measurable clinical characteristics of a patient, including hypoxemia severity, respiratory mechanics, imaging findings, laboratory parameters, and coagulation status; in this sense, ARDS itself constitutes a clinical phenotype [15]. A subphenotype represents a reproducible subgroup within that phenotype, defined by shared clinical and biological traits [15,148]. An endotype denotes a deeper level of classification, characterized by a specific underlying pathophysiological mechanism with potential therapeutic implications [15].

A fundamental advance in ARDS phenotyping occurred in 2014 with a study published in *The Lancet Respiratory Medicine* by Calfee et al. [6] through a secondary analysis of the ARMA and ALVEOLI trials using Latent Class Analysis (LCA). The hyperinflammatory group exhibited higher circulating inflammatory mediators (IL-6, IL-8, soluble tumor necrosis factor receptor-1 (sTNFR-1), and plasminogen activator inhibitor-1 (PAI-1)), greater vasopressor use, lower serum bicarbonate, and higher sepsis prevalence. Importantly, this subgroup had significantly higher mortality in both trials (44% vs. 23% in ARMA; 51% vs. 19% in ALVEOLI), revealing heterogeneity of therapeutic effect [6].

These findings were subsequently reproduced in independent cohorts. In the multinational LUNG SAFE study (*n* = 2813), approximately 74% of patients were classified as hypoinflammatory and 26% as hyperinflammatory [149]. The hyperinflammatory subphenotype was associated with higher 90-day mortality and fewer ventilator-free days. A differential response to PEEP was observed, with reduced mortality in hyperinflammatory patients receiving higher PEEP, while no significant effect was detected in the hypoinflammatory group. Similar reproducibility was demonstrated in the VALID (*n* = 452) and EARLI (*n* = 335) cohorts, where the hyperinflammatory subphenotype consistently showed higher in-hospital mortality. Parsimonious clinical models achieved strong discriminatory performance (AUC 0.92 in EARLI and 0.88 in VALID), indicating that risk stratification may be feasible using routinely available variables.

Evidence of phenotype-dependent therapeutic response was further supported in interventional trials. In FACTT (*n* = 1000), a significant interaction between phenotype and fluid strategy was identified (*p* = 0.0039) [150]. Conservative fluid management reduced mortality in hypoinflammatory patients, whereas liberal fluid administration was associated with lower mortality in the hyperinflammatory subgroup.

Similarly, in HARP-2 (*n* = 539), simvastatin showed no overall survival benefit in the primary analysis [12]. However, LCA again identified hypoinflammatory (65%) and hyperinflammatory (35%) subphenotypes, with a significant interaction between phenotype and treatment. The hyperinflammatory subgroup exhibited higher baseline mortality and fewer ventilator- and organ failure–free days, but demonstrated improved survival with simvastatin, whereas no benefit was observed in the hypoinflammatory group.

The reproducibility of this biological stratification has also been demonstrated beyond adult non-COVID ARDS. In pediatric ARDS (PARDS), Dahmer et al. [151] identified analogous hypoinflammatory (≈60%) and hyperinflammatory (≈40%) subphenotypes, with the latter associated with higher inflammatory markers, greater vasopressor use, increased severity, longer mechanical ventilation, and higher mortality.

In ARDS secondary to SARS-CoV-2 infection, Sinha et al. [152] similarly identified hypoinflammatory (77%) and hyperinflammatory (23%) subphenotypes. The hyperinflammatory group exhibited higher inflammatory and injury biomarkers, greater organ dysfunction, increased vasopressor requirement, and markedly higher 90-day mortality. A significant interaction with corticosteroid therapy was observed, with reduced mortality among hyperinflammatory patients receiving steroids, whereas corticosteroid use was associated with worse survival in the hypoinflammatory subgroup. Collectively, these studies demonstrate that biologically defined ARDS subphenotypes are reproducible across cohorts, age groups, and etiologies, and are associated with distinct prognostic profiles and differential therapeutic responses. A comparative summary of these findings is presented in Table 2.

Most ARDS phenotyping studies, including the seminal and validation analyses [6,12,150], have used blood plasma as the principal biological matrix. This approach reflects the systemic nature of the hyperinflammatory phenotype, as plasma biomarkers correlate with extrapulmonary organ dysfunction and severity scores such as SOFA and APACHE II [153,154]. The predominant analytical platform has been multiplex assays (e.g., Luminex), enabling simultaneous quantification of multiple inflammatory mediators [153,154,155]. However, plasma biomarkers do not directly represent the local inflammatory milieu of the alveolar compartment, which can be assessed through bronchoalveolar lavage fluid (BALF). In a multicenter study of 88 ARDS patients, Sathe et al. [154] compared plasma-defined hypoinflammatory and hyperinflammatory subphenotypes with BALF biomarker profiles. Minimal differences in BALF IL-6 and G-CSF levels were observed between systemic subphenotypes, and concordance between plasma and BALF classifications was low (κ = 0.07). When LCA was applied exclusively to BALF biomarkers, two distinct alveolar phenotypes emerged, independent of the systemic classification.

BALF Class 2 (72% of patients) was characterized by elevated total protein, higher neutrophil percentage, increased vWF and sPD-L1, and was associated with worse oxygenation (PaO_2_/FiO_2_ 152 vs. 202; *p* = 0.008), greater lung injury severity (54% vs. 20%; *p* = 0.004), and higher applied PEEP (10 vs. 5 cmH_2_O; *p* = 0.018). BALF Class 1 exhibited lower alveolar inflammatory burden and better respiratory parameters.

These data indicate a dissociation between systemic and alveolar biological stratification: plasma phenotypes reflect systemic inflammation and risk of multiorgan dysfunction, whereas BALF-derived phenotypes more directly capture the severity of pulmonary injury and gas exchange impairment. Table 3 summarizes the comparative characteristics of systemic (plasma) and alveolar (BALF) phenotyping approaches in ARDS.

### 6.2. Proteomic Subphenotypes and Therapeutic Response in ARDS

Lin et al. [16] evaluated high-density serum proteomics in 1048 patients with ARDS to determine whether early molecular profiling could identify clinically meaningful subphenotypes. Serum samples obtained within 72 h of diagnosis were analyzed using the Olink Explore 384 Inflammation panel, which quantifies 362 proteins spanning inflammatory mediators, immune receptors, cell injury markers, extracellular matrix regulators, and metabolic or inhibitory signaling molecules. Key analytes included IL-6, IL-8, Interleukin-1 receptor antagonist (IL-1RA), IL-10, Tumor Necrosis Factor Receptor Superfamily Member 14 (TNFRSF14), Interferon Gamma Receptor 1 (IFNGR1), DNA Fragmentation Factor Alpha (DFFA), Plasminogen Activator, Urokinase Receptor (PLAUR), Basigin (BSG/CD147), Agrin (AGRN), Coagulation Factor II Receptor (F2R), Enabled Homolog (ENAH), NEDD8 Ultimate Buster 1 (NUB1), Leukocyte-Associated Immunoglobulin-like Receptor 1 (LAIR1), Butyrophilin Subfamily 3 Member A2 (BTN3A2), and Lymphotoxin Beta Receptor (LTBR). LCA identified three reproducible phenotypes, designated C1, C2, and C3.

C1 corresponded to a hyperinflammatory profile characterized by activation of innate immune pathways, including TLR, Cyclic GMP-AMP Synthase–Stimulator of Interferon Genes (cGAS-STING), and Nucleotide-binding Oligomerization Domain-containing proteins 1 and 2 (NOD1/2). This phenotype showed marked overexpression of IL-6, IL-8, Triggering Receptor Expressed on Myeloid Cells 1 (TREM1), PLAUR, and TNFRSF14, all with *p*-values < 0.001. Clinically, C1 was associated with a higher proportion of non-aerated lung parenchyma, a median of 0 ventilator-free days with an interquartile range of 0–10, and the highest 90-day mortality at 72%. Compared with C2, the hazard ratio for death was 2.84, with a 95% confidence interval (CI) of 2.31–3.48 (*p* < 0.001).

C2 exhibited a profile dominated by relative immunosuppression and activation of anti-inflammatory and reparative pathways, including IL-10, IL-4 and IL-13, together with metabolic signaling involving Peroxisome Proliferator-Activated Receptor (PPAR), Farnesoid X Receptor/Retinoid X Receptor (FXR/RXR), Retinoic Acid Receptor (RAR), and sirtuins, all with *p* < 0.001. Clinically, this group had less organ dysfunction, a greater proportion of aerated lung tissue, a median of 20 ventilator-free days with an interquartile range of 0–28, and the lowest 90-day mortality at 41%.

C3 represented an intermediate immune-metabolic phenotype with partial activation of IL-6 and IL-17 signaling and mixed glycolytic and glycation signatures. It predominated in older patients, with a mean age of 71 years, and was associated with a 90-day mortality of 56%. Relative to C2, the hazard ratio (HR) for death was 1.70 (95% CI: 1.32–2.19).

Therapeutic heterogeneity was substantial. In 825 patients analyzed using Inverse Probability of Treatment Weighting-adjusted Cox models, glucocorticoid therapy was associated with reduced mortality in C1, with a HR of 0.56 (95% CI: 0.35–0.92; *p* = 0.022). In contrast, glucocorticoids were associated with increased mortality in C2, (HR = 1.77; 95% CI: 1.12–2.79; *p* = 0.014). No significant effect was observed in C3. The interaction between phenotype and corticosteroid therapy was statistically significant (*p* = 0.003).

A similar interaction was observed with ventilatory strategy. Among 607 mechanically ventilated patients, higher PEEP was associated with reduced mortality in C1 (HR = 0.62, 95% CI: 0.40–0.96; *p* = 0.032). In C2, higher PEEP was associated with increased mortality, (HR = 2.14, 95% CI: 1.19–3.87; *p* = 0.012) and no significant association was detected in C3. The interaction between phenotype and PEEP strategy was highly significant (*p* < 0.001).

According to above, early serum proteomic profiling identified three biologically distinct ARDS phenotypes with different clinical trajectories and opposing responses to corticosteroids and ventilatory strategies. These findings support the feasibility of biomarker-based therapeutic stratification in ARDS.

### 6.3. Biomarkers in ARDS

Biomarker research in ARDS has evolved from descriptive association studies to clinically oriented stratification strategies with predictive and therapeutic implications [23]. Subphenotype analyses derived from randomized trials and multicenter cohorts demonstrate that biological heterogeneity is measurable and clinically relevant [12,16]. The integration of biomarkers into adaptive or enrichment trial designs represents a rational approach to address therapeutic non-response and advance precision medicine in ARDS [16]. Given the complexity of ARDS pathophysiology, biomarkers are most informative when they reflect specific biological pathways rather than nonspecific inflammation.

#### 6.3.1. Inflammatory Markers

Systemic and alveolar inflammation are central determinants of ARDS heterogeneity [6,16]. Among circulating mediators, the IL-6 and IL-8 axis defines the hyperinflammatory phenotype identified by latent class analysis [6,12,16]. Persistent elevation of these cytokines, rather than baseline values alone, correlates with increased mortality, prolonged mechanical ventilation, and higher severity scores across classic and COVID-19-related ARDS cohorts [6,154,156,157,158]. For example, sustained IL-6 elevation at day 7 has shown discriminatory capacity for hospital mortality, underscoring the prognostic relevance of inflammatory persistence [157]. IL-8 correlates with APACHE II scores and ongoing alveolar inflammation, even under corticosteroid therapy, potentially explaining delayed ventilator liberation in selected patients [78,156].

Comparative analyses between COVID-19 and non-COVID ARDS indicate that systemic IL-6 concentrations are substantially lower than in bacterial sepsis, challenging the concept of a uniform “cytokine storm” and suggesting compartmentalization between lung and plasma [26,159]. This heterogeneity likely contributes to the context-dependent efficacy of IL-6 receptor antagonists, whose benefit appears modest and influenced by baseline inflammatory status and concomitant corticosteroid use [160,161,162].

Beyond IL-6 and IL-8, pathway-specific biomarkers provide incremental prognostic value. IL-18, a marker of NLRP3 inflammasome activation, identifies a high-risk subgroup even within the hypoinflammatory phenotype, with markedly increased short-term mortality and differential response to therapies such as statins [163]. IL-1RA despite its anti-inflammatory function, correlates with hypoxemia severity, prolonged ventilation, and mortality in both adult and pediatric populations, improving discrimination when combined with SOFA or APACHE II scores [164,165]. The TNF-α pathway, particularly its soluble receptor sTNFR-1, consistently predicts adverse outcomes [6]. Elevated or rising sTNFR-1 levels characterize hyperinflammatory trajectories associated with mortality exceeding 40 percent and greater need for advanced supportive therapies [166,167]. Conversely, low TNF-α concentrations define rapidly improving ARDS phenotypes with substantially lower mortality, a distinction relevant for trial enrichment strategies [168].

Beta-2 microglobulin (B2M), a marker of systemic immune activation and MHC class I turnover, has recently been associated with increased short-term mortality in sepsis-related ARDS, independently of conventional severity scores [169]. Elevated circulating levels correlate with multiorgan dysfunction and inflammatory burden, suggesting incremental prognostic value beyond classical cytokine profiling. Although external validation remains limited, B2M may represent an emerging marker of immune-driven risk stratification in biologically heterogeneous ARDS populations [169].

C-X-C motif chemokine ligand 16 (CXCL-16) has also emerged as an independent predictor of ARDS onset, mechanical ventilation requirement, and intensive care unit mortality in sepsis [165]. Early elevations correlate with disease severity and reflect fibroproliferative activity within the lung. Its prognostic accuracy improves when combined with biomarkers such as RAGE, Ang-2, and SP-D and integrated with clinical indices, supporting its role in multimarker risk stratification in sepsis-associated ARDS [165].

Among routinely available markers, the neutrophil-to-lymphocyte ratio (NLR) independently predicts mortality and progression, especially when assessed longitudinally. However, corticosteroid-induced neutrophilia may confound interpretation [170,171,172]. Large COVID-19 cohorts have proposed NLR thresholds to identify subgroups more likely to benefit from immunosuppression, illustrating its potential for risk-adapted therapy [173]. Markers such as circulating nucleated red blood cells (nRBCs), high-mobility group box 1 (HMGB1), and calprotectin or Calgranulin B (S100A8/A9) reflect advanced systemic injury and are associated with mechanical ventilation dependence, multiorgan failure, and high mortality, often indicating late-stage physiological decompensation [75,76,174,175,176].

Among currently validated biomarkers, soluble urokinase plasminogen activator receptor (suPAR) has the strongest evidence for therapeutic guidance. In the SAVE trial, a threshold ≥6 ng/mL identified patients at high risk of respiratory deterioration in whom Interleukin-1 blockade with anakinra significantly reduced progression to respiratory failure and decreased 30- and 90-day mortality [112]. This represents a model of biomarker-guided intervention with direct translational implications [177,178].

Overall, inflammatory biomarkers in ARDS provide maximal clinical value when interpreted dynamically and integrated with clinical severity indices. Their principal utility lies in biological stratification and identification of subgroups with divergent therapeutic responses, rather than isolated prognostication based on single measurements.

The main inflammatory biomarkers, their methodological context, biological matrices, cut-off values, and clinical utility in ARDS are summarized in Table 4.

#### 6.3.2. Alveolar–Epithelial Damage Biomarkers

Biomarkers of alveolar–epithelial injury reflect disruption of the alveolocapillary barrier, a structural determinant of persistent hypoxemia, pulmonary edema, prolonged mechanical ventilation, and mortality in ARDS [7,30,100,187,188,189,190,191].

sRAGE is the most validated epithelial biomarker. Individual patient meta-analysis data demonstrate an independent association between elevated baseline sRAGE and 90-day mortality (OR 1.18; 95% CI 1.01–1.38), regardless of ventilatory parameters [189]. sRAGE inversely correlates with alveolar fluid clearance, supporting its role as a functional marker of epithelial dysfunction [188]. Mendelian randomization suggests a causal relationship in sepsis-associated ARDS (OR up to 2.56) [30]. In COVID-19-related ARDS, levels > 3500 pg/mL identify markedly increased mortality risk (HR > 6), even when IL-6 loses prognostic discrimination under corticosteroid therapy [7,192].

SP-D derived from AEC2, shows prognostic value in pediatric ARDS but inconsistent mortality prediction in adult COVID-19 cohorts, suggesting greater utility for early risk assessment than outcome discrimination [100,101].

CC16 reflects epithelial permeability. Elevated day-1 plasma concentrations predict 90-day mortality with AUC ≈ 0.78, and values around 45 ng/mL discriminate mortality up to 50% versus <10% in low-level groups [33,190]. CC16 also identifies subgroups with differential response to fluid strategies, reinforcing its potential for therapeutic stratification [33,190].

Krebs von den Lungen-6 (KL-6) indicates sustained epithelial injury and early fibroproliferation. Persistently elevated levels (cutoffs 800–1450 U/mL) predict poor 28-day survival with AUC ≈ 0.78 [191]. In SARS-CoV-2 ARDS, KL-6 outperformed conventional inflammatory markers for severity prediction (OR 4.6) [193].

Emerging markers such as Transmembrane 9 Superfamily Member 1 (TM9SF1) show independent associations with severity (OR 2.43) and mortality (HR 2.27), but evidence remains observational and requires validation [74,194].

Table 5 summarizes the principal biomarkers of alveolar–epithelial damage in ARDS, including their exemplary studies, biological matrices and timing of measurement, cut-off values, and clinical utility for prognostic stratification, risk assessment, and therapeutic guidance.

#### 6.3.3. Endothelial Injury and Dysregulated Coagulation–Fibrinolysis

Endothelial dysfunction and coagulation imbalance integrate vascular permeability, microthrombosis, and fibrinolytic suppression, key contributors to ARDS heterogeneity and limited response to purely anti-inflammatory therapies [53,107,197,198,199]. From a clinical perspective, endothelial and hemostatic biomarkers capture dimensions of lung injury closely linked to prolonged mechanical ventilation, weaning failure, and the need for advanced support, including prolonged proning, renal replacement therapy, and vasopressor support [32,200,201].

Endocan (ESM-1) predicts ARDS development and mortality, with reported AUCs up to 0.93 in early risk stratification and adjusted HRs ≈ 1.3–1.4 for death in established ARDS [202,203,204].

Von Willebrand factor (VWF) is a robust marker of endothelial activation. In critical illness, levels exceeding 4–5 times normal are associated with a 9–10-fold increased mortality risk and AUCs up to 0.92, independent of inflammatory markers [199]. Ang-2 is among the most reproducible endothelial predictors of mortality (OR ≈ 1.7–1.9 per log increase) [32]. Temporal increases during early ICU stay strongly predict death (HR > 6; AUC > 0.90) and prolonged ventilation [205].

Ang-2 has also identified subgroups with differential responses to endothelium-targeted therapies such as statins or imatinib [195].

Syndecan-1 (SDC-1) reflects glycocalyx degradation and associates with fluid overload, worse oxygenation, and increased mortality (adjusted OR up to 7 in COVID-19 cohorts) [197,200,206,207]. Persistent elevated levels during the first week of mechanical ventilation identify patients with sustained endotheliopathy and a low probability of early pulmonary recovery [197].

Within the coagulation axis, soluble thrombomodulin (sTM) independently predicts mortality and fewer ventilator-free days (AUC ≈ 0.75–0.80) [198,201,208].

PAI-1 reflects hypofibrinolysis and correlates with severity and mortality (aOR 2–3), as well as weaning failure [53,107,209,210].

Citrullinated histone H3 (CitH3) integrates neutrophil extracellular trap formation, endothelial injury, and microvascular thrombosis, with high specificity for fatal outcomes [211,212].

Novel markers such as thymidylate synthase (TYMS), plasma renin, neural precursor cell expressed, developmentally downregulated 9 (NEDD9), and osteoprotegerin (TNFRSF11B) capture metabolic endothelial dysfunction and thrombo-inflammatory phenotypes, with strong effect sizes but limited prospective validation [27,51,90,213].

Collectively, endothelial–coagulopathic biomarkers provide independent prognostic information and identify biologically distinct endotheliopathy phenotypes.

Table 6 summarizes the principal biomarkers of endothelial injury and dysregulated coagulation–fibrinolysis in ARDS, including their exemplary studies, biological matrices and timing of measurement, cut-off values, and clinical utility for prognostic stratification, risk assessment, and therapeutic guidance.

#### 6.3.4. Extracellular Matrix Remodeling-Related Markers

ECM remodeling reflects irreversible structural injury, linking persistent inflammation, mechanical stress, and fibroproliferation [220,221,222]. Table 7 displays representative biomarkers of ECM remodeling in ARDS, including their exemplary studies, biological matrices and timing of measurement, cut-off values, and clinical utility for prognostic stratification, risk assessment, and therapeutic guidance.

Matrix metalloproteinase-3 (MMP-3) concentrations ≥ 18.4 ng/mL at day 3 predict 90-day mortality (48% vs. 4%; AUC 0.77), and dynamic increases correlate with fewer ventilator-free days [223].

Tissue inhibitor of metalloproteinases-1 (TIMP-1), particularly in women, predicts 30-day mortality for ARDS with AUC up to 0.87 [220]. Prognosis is more closely related to dysregulation of the MMP-9/TIMP-1 balance than to absolute levels [224,225]. Structural components including laminin, type IV collagen, hyaluronic acid, and procollagen type III N-terminal propeptide (PIIINP) reflect fibroproliferative activation and correlate with functional decline and mortality.

In severe ARDS requiring ECMO, PIIINP > 12.8 µg/L predicted mortality with AUC 0.87; progressive increases during ventilation were associated with HR ≈ 3 for 90-day death [17,221,226]. Urinary desmosine, a marker of elastolysis, independently predicts mortality and fewer ventilator-free days and is attenuated by lung-protective ventilation, directly linking mechanical stress to ECM degradation [222]. Of particular clinical relevance, elastolysis was attenuated by protective ventilatory strategies (low tidal volumes), establishing a direct link between mechanical stress, extracellular matrix destruction, and adverse outcomes [222]. ECM biomarkers therefore identify active remodeling phenotypes associated with irreversible injury and poor liberation from ventilation.

#### 6.3.5. Emerging Biomarkers

Emerging biomarkers extend beyond single pathogenic axes and reflect integrated systemic dysregulation [80,110,228,229,230,231,232].

Microvesicle-encapsulated miR-223 predicts 30-day mortality with AUC ≈ 0.70 and a fivefold increase in risk above defined thresholds, independent of physiological severity scores [230]. Transcriptomic panels discriminating sepsis-associated ARDS achieve AUCs ≈ 0.75–0.77 and reflect combined neutrophilic activation and adaptive immunosuppression [233]. Hepatic miR-122 independently predicts mortality (HR = 4.4; AUC = 0.78), highlighting lung–liver interaction [231]. Proteomic panels (including VCAM1, LDHB, MSN, LBP, MBL2) improve mortality prediction with AUC range of 0.80–0.89, outperforming isolated clinical models [80]. Metabolomic signatures using machine-learning approaches achieve AUC > 0.90 for early mortality prediction in COVID-19 ARDS [234]. Non-coding RNAs (circRNAs, lncRNAs) show high exploratory discrimination (AUC > 0.80–0.90) but lack standardization for clinical use [11,229,235]. Volatile organic compounds currently demonstrate limited incremental value (AUC ≤ 0.70) and remain exploratory [232].

Table 8 summarizes emerging biomarkers for ARDS, including their exemplary studies, biological matrices and timing of measurement, cut-off values, and clinical utility for prognostic stratification, systemic risk assessment, and precision medicine approaches.

## 7. Modern Diagnostic Imaging in ARDS and Therapeutic Perspectives

### 7.1. Advances in Monitoring and Diagnostic Imaging of ARDS

Prognostic assessment in ARDS has evolved beyond the PaO_2_/FiO_2_ ratio toward functional imaging and monitoring tools that characterize lung heterogeneity, ventilator–lung interaction, and vascular dysfunction [238,239,240,241]. These modalities provide clinically actionable information that directly informs ventilator titration, identification of phenotypes, prediction of weaning failure, and selection of advanced therapies [238,240,242,243].

#### 7.1.1. Individualizing PEEP and Reducing Ventilator-Induced Lung Injury

Electrical impedance tomography (EIT) has emerged as the most robust bedside tool for real-time ventilation monitoring and PEEP individualization. In a meta-analysis, EIT-guided PEEP improved compliance (+4.33 mL/cmH_2_O), reduced driving pressure (−1.20 cmH_2_O), decreased mechanical power (−1.99 J/min), and was associated with lower hospital mortality (RR 0.64) compared with conventional strategies [239]. Importantly, optimal PEEP determined by EIT differs from PEEP/FiO_2_ tables in more than 80% of patients, underscoring the marked interindividual variability in recruitability [60]. In addition to ventilation, EIT has demonstrated superiority over quantitative CT for stratifying the functional severity of ARDS. In patients with post-lung transplant ARDS, EIT identified significant increases in dead space, impaired ventilation/perfusion matching, and greater ventilatory inefficiency in the most severe cases—parameters that were not detected by static anatomical imaging [241]. By enabling regional ventilation assessment, EIT reduces exposure to injurious stress and strain and indirectly improves prognosis.

Esophageal pressure monitoring refines this approach by estimating transpulmonary pressure and titrating ventilation according to effective lung stress. Although EPVent-2 showed no overall mortality benefit, stratified analyses revealed significant 60-day mortality reduction in patients with lower systemic severity (HR 0.43), highlighting phenotype-dependent efficacy [244]. Similarly, driving pressure has consolidated its role as a key prognostic target; strategies maintaining ΔP ≤ 14 cmH_2_O significantly reduce 28-day mortality (HR ~0.26) compared with conventional protective ventilation [242].

Mechanical power integrates volume, pressure, flow, and respiratory rate into a single descriptor of energy delivered to the lung. Its normalization to dynamic compliance (MP/Cdyn) improves prognostic discrimination, with strong independent association with mortality (HR = 7.97; C-statistic 0.813) [245].

These parameters collectively reinforce that limiting dynamic stress, not simply improving oxygenation, is central to outcome modification.

#### 7.1.2. Phenotyping Lung Morphology and Recruitability

Lung ultrasound (LUS) has evolved into a quantitative bedside biomarker of severity and prognosis. The LUS score demonstrates high diagnostic accuracy versus CT (AUC ~0.88) and independently predicts mortality and prolonged ventilation [246,247,248]. Each incremental point increases in-hospital mortality risk by approximately 11% after adjustment [247,248]. Beyond severity stratification, LUS differentiates focal and non-focal phenotypes, informing PEEP responsiveness and recruitment strategies [249]. Its temporal evolution correlates with mechanical power (r ≈ 0.6) and discriminates survivors from non-survivors at 72 h (AUC ~0.84), supporting its role as a dynamic marker of ventilatory risk [240]. In pediatrics, elevated LUS values obtained within 12 h of admission predict prolonged invasive mechanical ventilation with an AUC > 0.9 [250].

#### 7.1.3. Identifying the Vascular and Edematous Phenotype

Dual-energy CT (DECT) provides insight into pulmonary perfusion abnormalities and microvascular dysfunction. Quantified perfusion defects ≥24% are independently associated with increased mortality, even without macroscopic embolism [251]. This vascular imaging phenotype helps explain severe hypoxemia disproportionate to respiratory mechanics and may identify patients who could benefit from intensified anticoagulation or early extracorporeal support [60,251,252,253].

Extravascular lung water (EVLW), measured by transpulmonary thermodilution and indexed to predicted body weight, objectively quantifies pulmonary edema. EVLWI and the oxygenation index independently predict 28-day mortality (combined AUC > 0.8), outperforming traditional severity definitions and supporting earlier selection of advanced support strategies [238,254].

#### 7.1.4. Predicting Weaning Failure and Respiratory Effort

Diaphragm electrical activity (EAdi) provides direct assessment of respiratory effort and neuromuscular coupling. Elevated EAdi during spontaneous breathing trials predicts extubation failure (AUC ~0.76), identifying patients who meet conventional criteria yet sustain excessive diaphragmatic workload [255]. In non-invasive ventilation, early reduction in transpulmonary pressure swings predicts treatment success with high accuracy (AUC 0.97), enabling early identification of patients at risk of failure [243].

#### 7.1.5. Artificial Intelligence and Multimodal Integration

Artificial intelligence (AI) enhances the prognostic value of imaging by converting radiological data into quantitative risk models. A meta-analysis of 33 studies reported pooled AUCs of 0.91 for ARDS development and progression prediction [256]. Multimodal models integrating imaging and clinical variables achieve AUCs up to 0.97 for predicting severe ARDS, invasive ventilation, and advanced life support requirements [257]. Although external validation and standardization remain necessary, AI represents a scalable tool for early risk stratification and resource allocation.

Collectively, modern imaging and monitoring tools move ARDS management toward functional, phenotype-guided ventilation and early identification of high-risk patients, with direct implications for mortality and duration of support.

### 7.2. Biological Basis and Pathophysiological Rationale of Therapeutic Strategies in ARDS

ARDS is characterized by diffuse alveolar–capillary injury, inflammatory amplification, endothelial dysfunction, pulmonary edema, and mechanical heterogeneity that predispose to VILI [22,258]. Mechanical ventilation therefore acts not only as supportive therapy but as a mechanobiological intervention capable of modulating disease progression [245,258].

#### 7.2.1. Mechanoprotection: Limiting Stress, Strain, and Energy Transfer

Low tidal volume ventilation (4–8 mL/kg PBW) remains the cornerstone of therapy and is associated with significant mortality reduction (RR ~0.79; 95% CI: 0.66–0.94) compared with higher tidal volumes [259]. Excessive tidal volumes amplify biotrauma, characterized by inflammatory mediator release, epithelial–endothelial barrier disruption, and systemic propagation of injury [258,260,261].

Beyond tidal volume alone, driving pressure and mechanical power better capture injurious load. Elevated plateau pressure (>26 cmH_2_O) or driving pressure (>15 cmH_2_O) is associated with diffuse inflammatory activation even in apparently well-ventilated regions, explaining why reduction of dynamic strain confers systemic benefit [258]. Mechanical power normalized to lung compliance further refines risk stratification and independently predicts mortality [245].

Prone positioning reduces regional stress concentration, improves ventilation homogeneity, lowers driving pressure, and decreases mechanical power. Its survival benefit, demonstrated in the PROSEVA trial, is attributed primarily to attenuation of VILI rather than oxygenation improvement alone [262]. Benefit appears greatest in early, highly recruitable phenotypes before irreversible fibroproliferation develops [263,264].

Extracorporeal membrane oxygenation (ECMO) allows near-complete unloading of the lung, facilitating ultra-protective ventilation and limiting ongoing mechanical injury. Evidence suggests greater benefit with early initiation and in non-fibrotic phenotypes [13,265].

#### 7.2.2. Preventing Patient-Induced Lung Injury

Neuromuscular blocking agents reduce excessive respiratory effort and prevent patient-induced lung injury (P-SILI). Although overall mortality benefit was not demonstrated in unselected populations, Bayesian analyses indicate a high probability of benefit in patients with elevated respiratory system elastance (≥2 cmH_2_O/(mL/kg)), with absolute risk reduction around 9% [14]. These findings support a phenotype-guided approach, particularly in early “stiff lung” presentations with high inspiratory effort [14,266].

#### 7.2.3. Modulating Inflammation and Endothelial Dysfunction

Pharmacologic strategies demonstrate that timing and biological phenotype are critical. Early corticosteroid administration, particularly in hyperinflammatory phenotypes, reduces mortality and increases ventilator-free days, whereas prophylactic or delayed use may be harmful [267,268]. In COVID-19-associated ARDS, targeted immunomodulation with IL-6 or JAK inhibitors reduced mortality and progression to invasive ventilation, especially in biomarker-selected hyperinflammatory patients selected by biomarkers such as PCR or suPAR [112,269,270,271]. Heparin, beyond anticoagulation, exerts anti-inflammatory and glycocalyx-protective effects and has been associated with improved survival in extrapulmonary sepsis-related ARDS at higher doses [18]. Conservative fluid strategies limit endothelial stress and increase ventilator- and ICU-free days [37].

#### 7.2.4. Regenerative and Emerging Approaches

Mesenchymal stem cell therapies aim to promote immunomodulation and alveolar–capillary repair through paracrine mechanisms. Early-phase studies suggest potential benefit in hyperinflammatory phenotypes, though further validation is required before routine implementation [272,273]. Additionally, recent single-cell transcriptomic analyses have delineated specialized alveolar endothelial subtypes, including aerocytes optimized for gas exchange and general capillary cells with vasomotor and progenitor functions [19,274]. These discoveries refine our mechanistic understanding of vascular heterogeneity and repair in acute lung injury and may be extrapolatable to ARDS, where endothelial diversity could influence both injury progression and recovery. Nevertheless, their clinical relevance for ARDS phenotyping and therapeutic targeting remains preliminary and will require rigorous translational validation.

Overall, therapeutic strategies in ARDS converge on a central principle: aligning the dominant biological and mechanical injury phenotype with targeted intervention. Moving beyond oxygenation-based severity classification toward mechanistic and phenotype-guided management represents a critical step toward precision medicine in ARDS [12,13,14].

## 8. Perspectives, Limitations, and Future Directions

ARDS is increasingly recognized as a biologically heterogeneous syndrome, which limits the effectiveness of uniform, syndromic therapeutic strategies. Integrating biological phenotyping and biomarker-informed stratification into clinical practice may improve prognostic precision, guide ventilatory and adjunctive therapies, and enable more rational patient selection for clinical trials. In this context, a next-generation therapeutic paradigm is likely to emerge not from a single pharmacologic agent, but from the integration of mechanical power–guided ultra-protective ventilation with real-time biological phenotyping. Aligning ventilatory energy load, driving pressure, and recruitability with inflammatory and structural phenotypes represents a biologically coherent strategy supported by consistent associations between mechanical stress metrics and mortality. Such an approach may allow personalization of PEEP titration, neuromuscular blockade, prone positioning, and extracorporeal support according to the dominant injury mechanism.

Regarding methodological strategy, this review has limitations inherent to its narrative design. The absence of systematic quantitative synthesis restricts formal assessment of bias and effect size, and heterogeneity in experimental models, biomarker platforms, and clinical definitions limits comparability across studies. Moreover, most mechanistic insights derive from observational or preclinical data, constraining causal inference and immediate clinical translation.

According to this literature review, future research should prioritize three major directions. First, prospective validation of real-time biological and mechanical stratification strategies is needed to determine whether phenotype-guided management improves outcomes compared with conventional syndromic approaches. Second, defining optimal therapeutic windows and phenotype-specific indications for immunomodulation, neuromuscular blockade, extracorporeal support, and vascular-targeted therapies remains a critical unmet need. Third, the development of reproducible multimodal models integrating imaging, biomarker panels, and artificial intelligence is essential to generate scalable tools capable of modifying mortality rather than simply improving prognostic discrimination.

## 9. Concluding Remarks

ARDS remains a highly lethal syndrome driven by the convergence of epithelial and endothelial injury, dysregulated inflammation, surfactant dysfunction, and profound alterations in pulmonary mechanics and gas exchange. However, accumulating evidence demonstrates that ARDS is not a single disease process but a biologically heterogeneous spectrum of injury patterns that evolve dynamically over time and determine prognosis and treatment response.

Secondary analyses of major ARDS network trials and large multicenter cohorts consistently identify reproducible hypoinflammatory and hyperinflammatory subphenotypes defined by differences in IL-6, IL-8, sTNFR-1, PAI-1, Protein C, and metabolic acidosis. These subphenotypes are associated with absolute mortality differences exceeding 25 percent and, critically, with opposite responses to PEEP strategy, fluid management, statins, and corticosteroid therapy. This biological heterogeneity provides a mechanistic explanation for the repeated failure of pharmacologic interventions applied indiscriminately to unselected ARDS populations. Among biomarkers, sTNFR-1, IL-6 and IL-8 dynamics, IL-18, and suPAR capture the inflammatory axis that defines therapeutic responsiveness, while sRAGE and CC16 reflect the structural burden of epithelial injury and impaired alveolar fluid clearance. The dissociation between plasma and bronchoalveolar lavage phenotypes further demonstrates that systemic inflammation and local alveolar damage represent complementary but distinct dimensions of ARDS biology that influence hypoxemia severity and ventilatory requirements. Proteomic approaches reinforce this concept by identifying molecular phenotypes capable of predicting opposite responses to corticosteroids and ventilatory strategies using a limited and clinically applicable biomarker panel.

The findings described above indicate that effective ARDS management requires moving beyond syndromic definitions toward biologically informed models that integrate inflammatory activity, structural lung injury, and compartmental phenotyping. Ultimately, the most promising advances in ARDS management are likely to arise from strategies that integrate control of mechanical energy transfer with biologically defined phenotypes, rather than from uniform application of isolated pharmacologic interventions.

## Figures and Tables

**Table 1:** Comparative diagnostic criteria for ARDS: Berlin definition (2012) vs. New global definition (2024).

Criteria	Berlin Definition (2012)	New Global Definition (2024)
**Timing**	Acute onset ≤ 7 days from event or new/worsening respiratory symptoms.	
**Origin of Edema**	Not primarily attributable to cardiogenic cause; if in doubt, perform echocardiogram or hemodynamic evaluation.	
**Chest imaging**	Bilateral opacities on X-ray or CT scan not explained by effusion, atelectasis, or nodule.	Bilateral opacities on chest X-ray, CT or ultrasound (B-lines/consolidation) not explained by effusion or atelectasis.
**Oxygenation**	PaO_2_/FiO_2_ ≤ 300 mmHg with PEEP ≥ 5 cmH_2_O (or CPAP ≥ 5).	PaO_2_/FiO_2_ ≤ 300 mmHg or SpO_2_/FiO_2_ ≤ 315 (if SpO_2_ ≤ 97%), with PEEP/CPAP ≥ 5 cmH_2_O or HFNO ≥ 30 L/min.
**Ventilatory support**	Requires invasive or non-invasive mechanical ventilation with PEEP ≥ 5 cmH_2_O.	Includes patients with HFNO ≥ 30 L/min, invasive or non-invasive ventilation (PEEP/CPAP ≥ 5 cmH_2_O).
**Limited resource context**	Not specified	According to the Kigali modification, it allows diagnosis with S/F without arterial blood gas analysis and without the need for a specific PEEP, provided the device and FiO_2_ are documented.
**Severity Category**	Mild: 200 < P/F ≤ 300 mmHg Moderate: 100 < P/F ≤ 200 mmHg Severe: P/F ≤ 100 mmHg	Mild: 200 < P/F ≤ 300 mmHg or 235 < S/F ≤ 315 * Moderate: 100 < P/F ≤ 200 mmHg or 148 < S/F ≤ 235 * Severe: P/F ≤ 100 mmHg or S/F ≤ 148 *

Data obtained of: [2,5]. ARDS: acute respiratory distress syndrome; CT: computed tomography; PEEP: positive end-expiratory pressure; CPAP: continuous positive airway pressure; HFNO: high-flow nasal oxygen; FiO_2_: fraction of inspired oxygen; PaO_2_: partial pressure of oxygen in arterial blood; SpO_2_: peripheral oxygen saturation measured by pulse oximetry; P/F: ratio of PaO_2_ to FiO_2_; S/F: ratio of SpO_2_ to FiO_2_. *: S/F values are approximately equivalent to P/F cut-offs but are only valid if SpO_2_ ≤ 97%.

**Table 2:** Clinical characteristics and outcomes of hypoinflammatory and hyperinflammatory ARDS subphenotypes.

Characteristic	Subphenotype 1 (Hypoinflammatory)	Subphenotype 2 (Hyperinflammatory)
**Prevalence**	~60–70%	~30–40%
**90-day mortality**	20–25%	40–55%
**Prognosis (free days)**	Ventilator-free days (VFD): 15–20 Organ failure-free days: 22–27	Ventilator-free days: 0–8 Organ failure-free days: 4–15
**Plasma biomarkers**	IL-6 ↓ IL-8 ↓ sTNFR-1 ↓ PAI-1 ↓ Protein C ↑/normal	IL-6 ↑ IL-8 ↑ sTNFR-1 ↑ PAI-1 ↑ Protein C ↓
**Serum bicarbonate**	Normal/high (≈22–26 mmol/L)	Low (≈18–20 mmol/L; ↑ metabolic acidosis)
**Vasopressor use at enrollment**	15–25%	60–70%
**Primary ARDS risk factor**	Trauma, aspiration, pneumonia predominant	Sepsis predominant (~50%)
**Response to PEEP (alveoli, lung safe)**	Similar mortality (32–34%)	Benefit with high PEEP (54% vs. 62%; *p* = 0.041)
**Response to fluid management (FACTT)**	Conservative strategy reduces mortality (18% vs. 26%)	Liberal strategy reduces mortality (40% vs. 50%)
**Response to Simvastatin (HARP-2)**	No benefit (28-day mortality ≈ 16–17%)	Significant benefit (28-day mortality: 32% vs. 45%; *p* = 0.008)
**Outcomes (Pediatrics)**	Mortality 2.2%; VFD ≈ 6.6 días; vasopressors 35%; sepsis 7%	Mortality 13.8%; VFD ≈ 10 días; vasopressors 80%; sepsis 39%.
**Outcomes (COVID-19)**	Mortality 48%; bicarbonate 21.5 mmol/L; vasopressors 80%; SOFA 9	Mortality 75%; Bicarbonate 16.1 mmol/L; vasopressors 99%; SOFA 12; corticosteroid interaction.

Data obtained from [6,12,149,150,151,152]. IL: Interleukin; sTNFR-1: soluble tumor necrosis factor receptor-1; PAI-1: Plasminogen activator inhibitor-1; Protein C: Protein C; VFD: ventilator-free days; SOFA: sequential organ failure assessment; PEEP: positive end-expiratory pressure. Arrows indicate direction of change: ↑ increased levels; ↓ decreased levels.

**Table 3:** Comparison of systemic (plasma) vs. alveolar (BALF) phenotyping in ARDS.

Characteristic	Systemic Phenotyping (Plasma)	Alveolar Phenotyping (BALF)
**Biological matrix**	Plasma (venous/arterial blood)	BALF obtained via bronchoscopy
**Underlying model**	LCA based on plasma biomarkers: Hypoinflammatory vs. Hyperinflammatory	LCA based on BALF biomarkers: BALF Class 1 vs. BALF Class 2
**Typical biomarkers**	IL-6, IL-8, sTNFR-1, PAI-1, Protein C, Bicarbonate	IL-6, vWF, sPD-L1, Total protein, % neutrophils, 25-Hydroxycholesterol
**Clinical correlation**	↑ Extrapulmonary organ dysfunction (SOFA, APACHE II, vasopressors)	↑ Severity of lung injury (PaO_2_/FiO_2_, LIS, ventilatory parameters, hypoxemia)
**Overlap between classes**	Minimal; both plasma phenotypes cluster into BALF Class 2	BALF Class 2 shows high alveolar inflammation regardless of plasma phenotype
**Representative findings**	↑ Mortality, ↑ systemic inflammation, ↑ multiorgan dysfunction	↓ Oxygenation, ↑ lung injury score, ↑ alveolar inflammation
**Therapeutic implication**	Suitable for systemic therapies (IV immunomodulators, fluid strategies, statins)	Suitable for lung-targeted therapies (inhaled anti-inflammatories, surfactant, local modulators)
**Limitations**	May not reflect alveolar inflammation; systemic bias	Invasive, dilution variability, small sample size; requires external validation

Data obtained from [6,154]. ARDS: Acute respiratory distress syndrome; BALF: bronchoalveolar lavage fluid; SOFA: sequential organ failure assessment; APACHE II: acute physiology and chronic health evaluation II; LIS: lung injury score; IL: interleukin; sTNFR-1: soluble tumor necrosis factor receptor-1; vWF: von Willebrand Factor; sPD-L1: soluble programmed death-ligand 1. Arrows indicate direction of change: ↑ increased levels or severity; ↓ decreased levels.

**Table 4:** Inflammatory Biomarkers for Prognostic and Phenotypic Stratification in ARDS.

Biomarker	Best Exemplary Study/Methodology	Biological Matrix/Time of Measurement	Cut-Offs/Levels	ARDS Usefulness
**IL-6**	Lin et al. [16] Multicenter, prospective cohort study in (*n* = 1048 patients with ARDS)	Serum; Within 72 h of diagnosis Other matrices studied: Plasma and BALF	Phenotype C1 (Hyper): Median 162.7 pg/mL. Phenotype C2 (Hypo/Repair): Median 26.1 pg/mL. Phenotype C3 (Intermediate): Median 52.9 pg/mL.	Molecular phenotyping and monitoring of treatment response with glucocorticoids, statins, biologics or PEEP [6,12,16,160,161]. Prognostic indicator of progression, severity, and mortality [156,158], identify the RIARDS [168], prediction of the need for and duration of mechanical ventilation [6], etiological comparison: patients with ARDS due to COVID-19 have lower systemic levels of IL-6 than those with bacterial ARDS [159].
**IL-8**	Alipanah-Lechner et al. [166] Secondary analysis of a randomized trial (*n* = 400 patients with severe COVID-19).	Plasma; Baseline/serial Other matrices studied: Serum and BALF	Subtype 2 (Worse prognosis): Median 14.8 pg/mL Subtype 1 (Better prognosis): Median 11.4 pg/mL In BALF, concentrations are extremely high (CARDS without steroids: 19,940 pg/mL [78]	Stratification in COVID-19 [166]. In BALF IL-8 is a robust predictor of mortality (AUC = 0.813) [179] reflecting the severity of the lung injury. Systemic levels are associated with prolonged mechanical ventilation [12] and significant positive correlation with higher APACHE II scores [156]. Combined use enhances prediction of fatal outcomes, and IL-8 helps distinguish hyperinflammatory ARDS from RIARDS [6,168].
**IL-10**	Smail et al. [180] Prospective case–control study (*n* = 240). Analyzed serum levels and SNPs in CARDS.	Serum and DNA; Upon hospital admission. Other matrices studied: Plasma, BALF, PBMCs	Severe (10.74 pg/mL), Moderate (6.56 pg/mL), Controls (1.44 pg/mL) In BALF, non-survivors showed 4.0 pg/mL vs. 2.4 pg/mL in survivors (*p* > 0.05, not significant) [179]	Elevated serum levels are associated with greater severity and mortality, while a genetic predisposition for high IL-10 production (−1082 G/G polymorphism) protects against the development of severe forms [180]. Clinically, its greatest utility lies in evaluating immune homeostasis, where an increase in the IL-10/IL-6 ratio indicates recovery and a favorable response to immunomodulators [112].
**IL-18**	Moore et al. [163] Secondary analysis of two randomized trials (SAILS *n* = 683, HARP-2 *n* = 511).	Plasma; Baseline Other matrices studied: Serum	High-risk cutoff: ≥800 pg/mL. SAILS Median: 554 pg/mL. HARP-2 median: 845 pg/mL.	Advanced risk stratification identifies hidden high-risk hypoinflammatory patients [163], independently predicts mortality, and monitors corticosteroid and oxygen therapy efficacy [172].
**IL-1RA**	Dahmer et al. [164] Prospective cohort study (BALI/RESTORE, *n* = 549 ventilated children). Longitudinal analysis.	Plasma; Day 0 (intubation) to Day 3. Other matrices studied: DNA, Serum, BALF, PBMCs	Very high values on day 0 (~10,000 pg/mL) and their persistence on day 1 (~1000 pg/mL) are associated with PARDS and worse outcomes	Independent indicator of mortality and worse clinical outcomes, including prolonged mechanical ventilation, in both adult sepsis and PARDS [164,165]. Distinction between direct and indirect lung injury in BALF [154]. Exogenous administration (Anakinra) has shown therapeutic efficacy in preventing progression to severe respiratory failure [112].
**TNF-α**	Yan et al. [181] Retrospective cohort study (Development *n* = 308, Validation *n* = 132). Development of a nomogram to predict ARDS in sepsis.	Serum; Within 24 h of sepsis diagnosis. Other matrices studied: Plasma, BALF	ARDS: Average 360.15 pg/L. Non-ARDS: Average 280.95 pg/L. BALF shows low levels (6–9 pg/mL) with no significant differences between healthy controls and patients with ARDS (COVID or Non-COVID) [78].	Independent predictor of ARDS development in sepsis patients [181] disease severity, progression to mechanical ventilation and mortality in COVID-19 patients [182,183]. Positive correlation with established clinical severity scores, such as APACHE II and SOFA, and other inflammatory biomarkers (such as suPAR and CRP) [177].
**sTNFr1**	Calfee et al. [6,12] ACC analysis of randomized trials (HARP-2, ALVEOLI, ARMA). *n* = 539 (HARP-2) and >1000 (others).	Plasma. Baseline (<36 h from diagnosis) Other matrices studied: Serum	Hyperinflammatory phenotype: Median 11,202 pg/mL (HARP-2)/4265 pg/mL (ALVEOLI). Hypoinflammatory phenotype: Median 3511 pg/mL (HARP-2)/3255 pg/mL (ALVEOLI).	The most robust and validated single biomarker for the molecular phenotyping of ARDS. It consistently identifies the “hyperinflammatory” phenotype (or C1/Subtype 2 phenotype in newer models) [12,16,166]. It is a predictor of significantly higher mortality in both classic ARDS and COVID-19. High levels of sTNFr1 predict a favorable response to specific therapies such as simvastatin and high PEEP strategies, interventions that are ineffective in patients with low levels (hypoinflammatory phenotype) [6,12].
**Ferritin**	Shakaroun et al. [184] Retrospective cohort study (*n* = 2265 hospitalized for COVID-19).	Serum; Admission (first 24h) and longitudinal (Day 1–4 in ICU). Other matrices studied: plasma	≥490 ng/mL acts as an independent predictor of mortality, ICU admission and need for mechanical ventilation (typically >380–500 ng/mL).	Severity and prognosis (especially associated with COVID-19 and pneumonia in the elderly): elevated levels on admission predict with high sensitivity 28-day mortality, need for mechanical ventilation, and ICU admission, outperforming traditional markers such as CRP or procalcitonin [184]. Its utility is enhanced when combined with clinical scores such as SOFA or CURB-65 [185].
**B2M**	Cui et al. [169] Retrospective cohort study (*n* = 257 adults with ARDS due to bacterial infection).	Serum; first 24 h after ARDS diagnosis	Optimal mortality cutoff: 4.6 mg/L (Median): Total: 4.7 mg/L; Non-survivors: 6.3 mg/L; Survivors: 3.7 mg/L	Elevated serum levels within the first 24 h in patients with sepsis-related respiratory distress reflect systemic inflammation, renal dysfunction, and hypoxemia severity [169]. This biomarker independently predicts 28-day mortality, with accuracy superior to PaO_2_/FiO_2_ and comparable to the SOFA score, supporting its role as an early risk stratification tool in critically ill patients [169].
**NLR**	Mehdi et al. [171] Retrospective cohort (*n* = 388 COVID-19).	Blood; On admission and days 3, 5, 7.	An elevated NLR on admission (≥3, median 8.9 vs. 4.2) and its serial increase in the first 7 days were independently associated with the development of ARDS	It predicts progression to ARDS and disease severity, distinguishing moderate/severe from mild forms [171]. it independently predicts 28-day mortality and shows greater consistency than CRP or IL-6, particularly under high-dose steroid therapy [157]. Accuracy improves when combined with platelet count (N/LPR) [170].
**nRBCs**	Schmidt et al. [174] Retrospective observational cohort study (*n* = 206 patients in ICU with ARDS due to COVID-19)	Peripheral blood (routine complete blood count); serial measurements during ICU stay.	Optimal mortality cutoff: >105/μL; Non-survivors: median of 355/μL; Survivors: median of 20/μL. nRBC > 10,000/μL was associated with 100% mortality. The maximum value reached during the stay was the key predictor, not the value at admission.	nRBCs act as a late “alarm” marker of severe bone marrow dysfunction driven by hypoxemia and systemic inflammation. Their levels peak after other biomarkers and clinical scores, often preceding fatal outcomes. nRBC positivity is an independent predictor of mortality, ventilation duration, and hospital stay, and its combination with SOFA > 8 markedly enhances the accuracy of death prediction beyond clinical scores alone [174].
**suPAR**	Chen et al. [177] Case–control study (*n* = 57 Sepsis-ARDS vs. 58 Sepsis no-ARDS).	Serum; within 24 h of the onset of ARDS or admission to ICU. Other matrices studied: plasma, BALF	Sepsis-ARDS (15.17 ng/mL) vs. Sepsis without ARDS (13.14 ng/mL). Cutoff Mortality: 17.38 ng/mL BALF: There were no differences between ARDS and No-ARDS (~2.5–2.7 ng/mL) [178]	Progression to respiratory failure and therapeutic guidelines: Plasma levels ≥ 6 ng/mL in COVID-19 pneumonia predict severe respiratory failure and allow for preventive intervention with Anakinra [112]. Independent predictor of ARDS development and mortality in sepsis, correlating with overall severity (APACHE II/SOFA) and inflammatory burden [177]. Specific indicator of fungal superinfection (aspergillosis) [178].
**Calprotectin** **(S100A8/A9)** **Calgranulin B (S100A9)**	Kassianidis et al. [76] Prospective cohort (*n* = 181) and clinical trial (SAVE-MORE).	Serum; Admission and serial (Day 4, 7). Other matrices studied: Whole blood, Lung tissue, BALF.	Risk cutoff: >7.8 µg/mL. Significant increase by day 7 in those who progress to ARDS/MV.	It robustly predicts progression to critical ARDS, the need for mechanical ventilation, and mortality [76]. Elevated serum and BALF levels of S100A9 (Calgranulin B), a calprotectin-like alarmin, are linked to poor long-term survival in pulmonary fibrosis. Inhibition with paquinimod has demonstrated efficacy in animal models of lethal coronavirus pneumonia, improving survival and attenuating fibrotic progression [186]. These findings highlight the therapeutic potential of targeting the S100A8/A9–TLR4 axis to treat severe ARDS and reduce its fibrotic sequelae [76,109,186].
**CXCL-16**	Villar et al. [165] (GEN-SEP Network) Multicenter observational study (*n* = 232 septic patients, 72 with ARDS).	Serum; Samples obtained within the first 24 h of sepsis diagnosis	Total Sepsis: 4255 pg/mL; Sepsis with mechanical ventilation: 5020 pg/mL; Sepsis without mechanical ventilation: 2985 pg/mL. Cutoff for predicting mortality in the ICU: Total Sepsis: ≥4424 pg/mL; Sepsis with mechanical ventilation: ≥4639 pg/mL	An independent and strong predictor of both the onset and severity of ARDS, the need for mechanical ventilation, and intensive care unit mortality among patients with sepsis. It reflects the proliferation of fibroblasts and collagen production, and therefore pulmonary fibrosis. Its clinical utility is enhanced when incorporated into multi-marker panels (including RAGE, Ang-2, and SP-D) and combined with variables such as PaO_2_/FiO_2_ or the APACHE II score, yielding diagnostic and prognostic accuracy superior to that of isolated clinical markers [165].

ARDS, acute respiratory distress syndrome; CARDS, COVID-19-associated ARDS; RIARDS, Rapidly Improving ARDS; BALF, bronchoalveolar lavage fluid; IL, interleukin; IL-1RA, interleukin-1 receptor antagonist; TNF-α, tumor necrosis factor-α; sTNFR-1, soluble TNF receptor-1; HMGB1, high-mobility group box-1; suPAR, soluble urokinase plasminogen activator receptor; NLR, neutrophil-to-lymphocyte ratio; nRBC, nucleated red blood cells; APACHE II, Acute Physiology and Chronic Health Evaluation II; SOFA, Sequential Organ Failure Assessment; MV, mechanical ventilation. Associations reflect clinical correlations (severity, mortality, MV duration or failure) and do not imply causality.

**Table 5:** Alveolar–epithelial damage biomarkers for prognostic and phenotypic stratification in ARDS.

Biomarker	Best Exemplary Study/Methodology	Biological Matrix/Time of Measurement	Cut-Offs/Levels	ARDS Utility
**sRAGE**	Jabaudon et al. [189] A meta-analysis of individual data (*n* = 746)	Plasma; Baseline. Other matrices studied: serum and BALF	Higher levels in non-survivors (median ~4335 pg/mL) vs. survivors (~3198 pg/mL) BALF: up to 30–100 times higher. The reported mean baseline level was 154,734 ± 217,417 pg/ML [188]	It predicts 90-day mortality independently of clinical severity and ventilatory parameters [189]. It predicts the decline in AFC [188]. It identifies hyperinflammatory phenotypes and severe degrees of pulmonary edema (correlation with RALE score) [7]. Genetic evidence suggests that it is not only a marker of damage, but a causal factor in the pathogenesis of ARDS [30].
**SP-D**	Villar et al. [165] (*n* = 232) adults with sepsis (152 on mechanical ventilation, 72 with ARDS). Multicenter prospective observational study (GEN-SEP).	Serum; for the diagnosis of sepsis (<24 h). Other matrices studied: plasma	Pulmonary Sepsis: Median 8.03 ng/mL Extrapulmonary Sepsis: Median 4.46 ng/mL ARDS vs. Non-ARDS: Significantly higher levels in ARDS.	It effectively distinguishes between sepsis of pulmonary and extrapulmonary origin [165]. It is part of a panel (along with RAGE, Ang-2, and CXCL16) used to predict the development of ARDS [165]. Elevated levels are independently associated with severe PARDS, higher mortality, longer duration of mechanical ventilation, and longer ICU stays [100]. It identifies a specific subphenotype (epithelial damage with systemic inflammation and endothelial dysfunction) that responds favorably to imatinib treatment (reduced mortality) [195]. It correlates positively with the severity of ARDS in COVID-19 [101]. Levels are consistently higher in direct ARDS compared to indirect ARDS. It has been used to predict mortality in several cohort studies [187].
**CC16**	Almuntashiri et al. [33] (*n* = 100) (ARDS) Secondary analysis of biomarkers from a multicenter RCT External Validation (ALTA Trial)	Plasma; Baseline. Other matrices studied: serum	Cut-off point: 45 ng/mL.	High levels on Day 1 strongly predict 90-day mortality (AUC 0.78) [190]. Subphenotyping suggests a potentially better response to fluid-conservative strategies in patients with high CC16 levels [190]. Levels ≥ 45 ng/mL are associated with higher 90-day mortality, fewer ventilator-free days, and fewer organ failure-free days [33]. This indicates specific damage to club cells (bronchioles) and increased epithelial permeability [196]. Extracellular vesicles containing CC16 have been shown to reduce inflammation in mouse models [196].
**KL-6**	Han et al. [191] (*n* = 50) 23 intrapulmonary ARDS and 27 extrapulmonary. Retrospective observational study, kinetic monitoring (7 days).	Serum; Admission, day 3 and day 7.	Severity: 335 U/mL predicts a severe outcome (ICU admission/Ventilation/Death) with an OR of 4.642.	Levels above the cutoff point predict poor 28-day survival. Peak levels are higher and occur later in intrapulmonary versus extrapulmonary non-survivors [191]. This identifies patients at risk of severe illness or death upon admission. Elevated levels are associated with the need for mechanical ventilation and ECMO [193].
**TM9SF1**	Cao et al. [74] (*n* = 239) ARDS (123 severe, 116 non-severe) + 52 healthy controls. Prospective observational cohort.	RT-qPCR in PBMCs (Peripheral blood mononuclear cells); Admission: Within 24 h of admission	Severity Prediction: Cutoff point > 0.07 Mortality Prediction: Cutoff point > 0.15 Severe ARDS: 0.21 ± 0.03. Non-severe ARDS: 0.08 ± 0.02 Healthy controls: 0.06 ± 0.01.	It predicts severity (OR 2.43) and mortality (HR 2.27) independently of age and comorbidities. It has better predictive performance (AUC 0.871 for severity) than traditional clinical markers such as CRP, D-dimer, and SOFA. The nomogram model integrates age, D-dimer, and CRP/NLR to calculate individualized risk [74].

ARDS, acute respiratory distress syndrome; sRAGE, soluble receptor for advanced glycation end-products; SP-D, surfactant protein D; CC16, club cell secretory protein; KL-6, Krebs von den Lungen-6 (MUC1); TM9SF1, transmembrane 9 superfamily member 1; BALF, bronchoalveolar lavage fluid; AFC, alveolar fluid clearance; RALE, Radiographic Assessment of Lung Edema; AUC, area under the ROC curve; HR, hazard ratio; OR, odds ratio. Associations denote epithelial injury burden, alveolar–capillary permeability, or repair failure and reflect clinical correlations (severity, mortality, MV duration or failure); causality cannot be inferred.

**Table 6:** Endothelial injury and dysregulated coagulation–fibrinolysis biomarkers for prognostic and phenotypic stratification in ARDS.

Biomarker	Study/Methodology	Biological Matrix/TM	Cut-Offs/Levels	ARDS Utility
**ESM-1**	Behnoush et al. [204]. *n* = 1058 (14 studies). Systematic review and meta-analysis.	Plasma/Serum; various times (in other studies measured at diagnosis).	~2–20 ng/mL, with consistently higher in non-survivors and in patients with progression of RF	Elevated levels at diagnosis are independently associated with mortality and multiple organ dysfunction (shock, renal failure) [203,204]. Low levels on admission in severe sepsis predict the early development of ARDS (72 h) [202]. In severe pneumonia, high levels are an independent risk factor for developing ARDS, contrasting with the sepsis profile [214]. Variability in measurement time [215].
**VWF**	Philippe et al. [199] *n* = 208 (COVID-19) Comparison of critical and non-critical cases. Cross-sectional, two-center study.	Plasma (platelet-poor); admission (≤48 h).	>423% predicts mortality (AUC = 0.92). Median in critical: 507% and non-critical: 288%.	The best predictor of in-hospital mortality from COVID-19 and ARDS. High levels and excess HMWM indicate microthrombosis and severe endothelial damage [199]. Elevated levels are associated with mortality and duration of mechanical ventilation [199,216]. Higher in ARDS of direct cause. Correlates with the Ventilatory Ratio (dead space) [51]. Elevated levels are associated with major thrombotic events and severe ARDS, indicating fibrinolytic suppression and endotheliopathy [53]. Consistently elevated in the Hyperinflammatory ARDS subphenotype [10].
**ANG-2**	Rosenberger et al. [32] *n* = 757 (267 with ARDS). Prospective cohort (EARLI), retrospective analysis. Patients with sepsis in ICU/ER	Plasma; Baseline (<24 h of admission to ICU). Other matrices studied: serum	Median in those who developed ARDS: 7577 pg/mL vs. Non-ARDS: 6032 pg/mL.	Predictor of ARDS and Mortality: Elevated levels predict the development of ARDS in sepsis. Associated with 30- and 60-day mortality in patients receiving corticosteroids, reflecting persistent endothelial damage [32,51]. The dynamic change is greater than the isolated baseline value. Early elevation predicts in-hospital death (HR 6.69); persistent elevation predicts “unresolved pulmonary condition” (fibrosis/chronic damage) [205]. It is a predictor of vasoplegia and shock [32].
**SDC-1**	Murphy et al. [200] *n* = 262 (Severe Sepsis). Retrospective observational (VALID study).	Plasma; admission and day 2 of ICU.	Global median ≈ 84 ng/mL in patients without vasopressors vs. 157 ng/mL in those requiring vasopressors	Persistently elevated levels are associated with the development of ARDS, worse oxygenation, fewer ventilator-free days, and cumulative positive fluid balance [197]. Independently associated with 60-day mortality (aOR = 8.0) [51]. The only biomarker correlated with the Ventilatory Ratio (dead space) at baseline and on day 3 [51]. Predicts ARDS specifically in non-pulmonary sepsis (indirect injury). Associated with in-hospital mortality and extrapulmonary organ failure (liver, kidney, coagulation) [200].
**sTM**	Liu et al. [198] *n* = 1992 (13 studies). Systematic review and meta-analysis.	Plasma/Serum; upon admission or diagnosis. Other matrices studied: Pulmonary Edema Fluid	SMD 1.47 (Non-survivors vs. Survivors). Combined AUC: 0.78.	Elevated levels predict hospital mortality regardless of severity (OR = 2.126). It improves the prediction of the APACHE III score [208]. Useful for risk stratification, although less predictive in direct ARDS (pneumonia) vs. indirect ARDS (sepsis) [198]. High levels in PARDS are associated with 90-day mortality, worsening oxygenation index (OI), and extrapulmonary multi-organ failure [201]. sTM is released locally in the damaged lung (epithelium/endothelium). High levels of edema are associated with death and fewer ventilator-free days [217]. In patients treated uniformly with corticosteroids/tocilizumab, sTM did not significantly discriminate mortality, suggesting that anti-inflammatory therapy may attenuate its predictive value [51].
**PAI-1**	Baycan et al. (2023) [210] *n* = 71 (hospitalized COVID-19 patients) + 20 controls. Retrospective/Cross-sectional.	Serum; upon admission. Other matrices studied: plasma, BALF	>10.2 ng/mL predicts mortality Mean Non-Survivors: 14 ng/mL vs. Survivors: 5 ng/mL.	Elevated levels are associated with severe ARDS, major thrombotic events, and fibrinolytic suppression [53]. It is an independent predictor of mortality and severity. It correlates strongly with the CT severity score (CT-SS) [210]. Levels are significantly higher in patients with greater hypoxemia, correlating with respiratory severity [107]. It is a distinctive marker of the hyperinflammatory subphenotype [10]. PAI-1 is present in the injured lung, forming complexes with factor VII activating protease (FSAP), inhibiting its protective activity [209].
**CitH3**	Tian et al. [212] *n* = 160 (102 with septic shock, 32 with non-infectious shock, 26 healthy). Prospective, observational, multicenter cohort study. (Applicable to human diseases associated with acute NETosis, such as ARDS).	Serum; on admission (0 h), 24 h and 48 h.	>39 pg/mL: Separates sepsis from healthy individuals (PPV 98%). >66 pg/mL: Separates septic shock from non-infectious shock (PPV 89.5%). Median Sepsis: 101.5 pg/mL vs. Healthy Individuals: 8 pg/mL.	It distinguishes septic from non-septic shock better than procalcitonin. It correlates with respiratory SOFA (r = 0.31) and PAD2/PAD4. High levels at 24–48 h predict 90-day mortality. CytH3 levels are negatively correlated with oxygenation (SpO_2_/FiO_2_), suggesting a direct role in respiratory dysfunction and pulmonary microthrombosis. It is a reliable blood marker for the diagnosis and treatment of endotoxic shock, a precursor to acute lung injury.
**TYMS**	Li et al. [90] *n* = 47 (ARDS) vs. *n* = 5 (Control) in training set; external validation with *n* = 15. In vivo validation in young vs. old C57BL/6 mice with LPS-induced ARDS (*n* = 6 per group)	mRNA (transcriptome) in tracheal aspirate (humans) and lung tissue (mice)	A high-expression group and a low-expression group were identified. As a result, 582 genes showed upregulation and 544 genes showed downregulation.	Diagnosis and Subphenotype of Aging: Identifies ARDS with high accuracy and distinguishes ARDS from sepsis. Marker of endothelial repair capacity; inadequate induction in the elderly suggests a worse prognosis due to impaired vascular regeneration [90].
**Renin**	Bellomo et al. [218] *n* = 255 (Vasodilator shock, ARDS subgroup). Post hoc analysis of the ATHOS-3 trial (RCT).	Serum; baseline (before drug) and at 3 h. Other matrices studied: plasma	Median population: 172.7 pg/mL (~3 × the upper normal limit). Normal range: 2.13–58.78 pg/mL.	Elevated levels indicate ACE deficiency (endothelial damage). Patients with elevated renin levels (>median) treated with angiotensin II had lower mortality (HR 0.56) compared to placebo [218]. Elevated levels on day 3 are associated with a sixfold increased risk of death at 30 days (OR = 6.85). A sustained elevation (from day 0 to 3) indicates the greatest risk of death [213].
**NEDD9**	Alladina et al. [51] *n* = 69 (intubated patients with ARDS due to COVID-19). Prospective observational cohort. All received corticosteroids.	Plasma; Day 1 of admission to ICU (within 24 h of intubation). Other matrices studied: Lung Tissue (Arteriolar Endothelium), Cell Lysates, Plasma.	Median Non-Survivors: 8.4 ng/mL vs. Survivors: 6.9 ng/mL (*p* = 0.0025).	It was independently associated with 60-day mortality (adjusted OR = 9.7), surpassing inflammatory markers in immunomodulated patients [51]. It is upregulated in the pulmonary arteriolar endothelium in lethal ARDS and colocalizes with intraluminal microthrombi, suggesting a direct role in immunothrombosis [51]. It is a key mediator of platelet-endothelium adhesion under hypoxic conditions. Its inhibition reduces platelet aggregate formation and acute pulmonary hypertension [25]. Persistently elevated levels are inversely associated with pulmonary microvascular perfusion and diffusing capacity (DLCO), indicating chronic endothelial dysfunction following acute injury [219].
**TNFRSF11B**	Zhang et al. [27] *n* = 50 (25 Sepsis-ARDS vs. 25 Healthy Controls). Human observational study with experimental validation in vivo (LPS mice) and in vitro (HUVECs). Analysis by Olink proteomics and ELISA.	Plasma; at admission (and in post-induction animal models with LPS).	ELISA: 0.76 ng/mL. AUC: 0.9600. Significantly higher levels in Sepsis-ARDS (~2.5 ng/mL mean) vs. Controls (~0 ng/mL).	Excellent predictive capacity for Sepsis-ARDS (AUC > 0.95). Indicates severe endothelial dysfunction: glycocalyx damage, disruption of cell junctions, and alteration of water channels, worsening pulmonary edema [27].

TM: time of measurement; ARDS, acute respiratory distress syndrome; ESM-1, endothelial cell–specific molecule-1 (endocan); vWF, von Willebrand factor; HMWM, high-molecular-weight multimers; RAAS, renin–angiotensin–aldosterone system; Ang II, angiotensin II; NEDD9, neural precursor cell expressed, developmentally down-regulated 9; TNFRSF11B, osteoprotegerin; ANG-2, angiopoietin-2; ANG-1, angiopoietin-1; SDC-1, syndecan-1; sTM, soluble thrombomodulin; PAI-1, plasminogen activator inhibitor-1; CitH3, citrullinated histone H3; NETs, neutrophil extracellular traps; Reported associations reflect endothelial activation, glycocalyx disruption, microthrombosis, or fibrinolytic suppression and correlate with severity, mortality, and MV-related outcomes; causality cannot be inferred.

**Table 7:** Extracellular matrix remodeling biomarkers for prognostic and phenotypic stratification in ARDS.

Biomarker	Best Exemplary Study/Methodology	Biological Matrix/Time of Measurement	Cut-Offs/Levels	ARDS Utility
**MMP-3**	Jones et al. [223]. *n* = 100 (ARDS patients, ALTA trial) + 20 healthy controls. Secondary analysis from an RCT.	Plasma; Day 0 (randomization) and Day 3.	Day 3: Optimal cutoff ≥ 18.4 ng/mL. Levels: Non-survivors (26.4 ng/mL) vs. Survivors (13.4 ng/mL) on Day 3.	Elevated levels on day 3 predict 90-day mortality (AUC 0.77). Associated with fewer ventilator-free and ICU-free days. Differentiates ARDS from healthy controls (AUC = 0.86) [223]. Elevates significantly and is associated with progression of severity in patients with COVID-19 [224].
**TIMP-1**	Almuntashiri et al. [220] *n* = 100 (ARDS patients from the ALTA trial) + 20 controls (RCT).	Plasma; Day 0 (assay randomization).	Cutoff (Women): ≥159.7 ng/mL for mortality. Levels: ARDS (132.5 ng/mL) vs. Normal (45.8 ng/mL).	It predicts 30- and 90-day mortality with high accuracy in women (AUC = 0.87). It was associated with fewer ICU- and ventilator-free days [220] and differentiated between ARDS and non-ARDS. It correlates positively with CT damage score, ferritin, and D-dimer. Levels decrease during recovery [225] and correlated positively with SOFA score and negatively with oxygenation (PaO_2_/FiO_2_). It indicates proteolytic imbalance [224].
**MMP-9**	Zingaropoli et al. [225] *n* = 129 COVID-19 (60 ARDS, 69 Non-ARDS) + 53 healthy. Longitudinal observational.	Plasma; Admission (Baseline) and 3 months post-hospital discharge.	Basal Levels: ARDS: 785 ng/mL Non-ARDS: 489 ng/mL Healthy: 287 ng/mL.	Higher enzyme levels and activity in ARDS vs. non-ARDS patients. Positively correlated with neutrophils and CRP, and negatively with PaO_2_/FiO_2_. Increased during the recovery phase, suggesting a role in repair [225]. Negatively correlated with the PaO_2_/FiO_2_ ratio in patients who developed ARDS, reflecting ALI. Altered levels were associated with an increased risk of in-hospital death. Heterogeneity in response to severity [224].
**Laminin**	Yu et al. [226] *n* = 162 (Patients with post-COVID pulmonary fibrosis) + 160 healthy controls. (RCT).	Serum; During post-infection follow-up (range 4–156 weeks).	Levels (Mean): Controls: ~58–70 ng/mL Deceased: 152.98 ± 50.47 ng/mL Survivors: 103.00 ± 43.27 ng/mL	Higher levels were found in patients who died within one year of follow-up (*p* = 0.016). Positive correlation with the HRCT and a negative correlation with pulmonary function (FVC% and DLCO%). It distinguishes between acute exacerbation (135.8 ng/mL) and stable disease (102.7 ng/mL) [226]. Its elevation in pulmonary fluids indicates direct damage to the basement membrane and extracellular matrix during ARDS [221].
**Desmosine**	McClintock et al. [222] *n* = 579 (subset of the ARDS Network trial of 861 patients).	Urine; Day 0 (Basal), Day 1 and Day 3.	Mean levels: 129 pmol/mg creatinine (vs. ~28 controls). VT 6 mL/kg group: 94 pmol/mg VT 12 mL/kg group: 98 pmol/mg	Higher baseline levels are independently associated with increased mortality. The increase in desmosine is significantly attenuated with low tidal volume ventilation (6 mL/kg), indicating less matrix damage. High levels correlate with fewer ventilator-free days and fewer organ failure days [222].
**PIIINP**	Yang et al. [227] *n* = 420 (COVID-19: 243 mild, 177 severe). Retrospective.	Serum; Upon hospital admission. Other matrix studied: Plasma, BALF	The text does not explicitly state the numerical cut-off concentration value for PIIINP in isolation. AUC Combined (with HA): 0.826 for predicting severity. Positive correlation with CRP, D-dimer, LDH.	Elevated levels distinguish severe from mild cases. They correlate with systemic inflammation, myocardial damage, and low oxygen saturation [227]. Levels > 12.8 µg/L at ECMO initiation predict death (AUC = 0.87, Sensitivity = 90%). They indicate active fibroproliferation associated with a poor prognosis [221]. Longitudinal (trajectory) increases in bronchoalveolar lavage (BAL) predict 90-day mortality [17]. Relatively high levels are associated with worse long-term lung function (DLCO/FVC). Persistently elevated levels are associated with greater fibrotic extent on HRCT, worse diffusion capacity (DLCO), and higher one-year mortality [226].

ARDS, Acute Respiratory Distress Syndrome; ALTA trial, Assessment of Low Tidal Volume and Elevated PEEP in Acute Lung Injury Trial; RCT, Randomized Controlled Trial; MMP-3, Matrix metalloproteinase-3 (stromelysin-1); TIMP-1, Tissue inhibitor of metalloproteinases-1; CT, Computed Tomography; SOFA, Sequential Organ Failure Assessment; PaO_2_/FiO_2_, Arterial oxygen tension to inspired oxygen fraction ratio; MMP-9, Matrix metalloproteinase-9 (gelatinase B); CRP, C-reactive protein; HRCT, High-resolution computed tomography; FVC, Forced Vital Capacity; DLCO, Diffusing capacity of the lung for carbon monoxide; VT, Tidal Volume; PIIINP, N-terminal propeptide of type III procollagen; BALF, Bronchoalveolar lavage fluid; HA, Hyaluronic acid.

**Table 8:** Emerging multimodal and systems-level biomarkers for prognostic and phenotypic stratification in ARDS.

Biomarker	Best Exemplary Study/Methodology	Biological Matrix/Time of Measurement	Cut-Offs/Levels	ARDS Usefulness
**MV-miR-223**	Almuntashiri et al. [230] *n* = 100 patients with ARDS (vs. 20 healthy controls). ROT from a randomized clinical trial	Human plasma (filtered to 0.8 µm to isolate MVs); day of randomization	Levels (Median): ARDS: 1.649 pg/mL Control: 0.655 pg/mL (*p* = 0.0003). Cut-off Mortality 30 days: 2.413 pg/mL.	It significantly distinguishes between patients with ARDS and healthy controls. High levels predict higher 30-day mortality (AUC = 0.7021). There is a significant negative correlation with ICU-free days, ventilator-free days, and organ failure. Higher levels are observed in infectious etiologies (sepsis/pneumonia) versus non-infectious etiologies.
**Multigene transcriptomic signatures**	Wei et al. [110] *n* = 196 (ARDS vs. Controls) and external validation. WGCNA and machine learning (SVM, Random Forest, Neural Networks).	Whole blood; Day 0 and Day 7 post-admission.	*LCN2*, *STAT3*, *SOCS3* upregulation; *AIF1L*, *SDHD* downregulation. AUC > 0.80 in training and validation cohorts for ARDS.	It identifies shared biomarkers between ARDS and sepsis-induced cardiomyopathy (SIC), suggesting common mechanisms of inflammation and mitochondrial dysfunction [110]. It effectively distinguishes between patients with sepsis alone and those who have developed sepsis-induced ARDS, reflecting immune dysfunction and neutrophil activation [233]. It predicts the onset of ARDS in septic patients with high accuracy (AUC = 0.86) [236].
**miR-122**	Rahmel et al. [231] (*n* = 119) patients with ARDS vs. 20 controls. Retrospective analysis of prospectively collected data and samples RT-qPCR	Human serum; Within the first 24 h of admission to the ICU (before therapies such as ECMO)	Levels 20 times higher in non-survivors vs. controls. Five times higher in non-survivors vs. survivors (*p* = 0.003). Cut-off 30-day mortality: Relative expression (2^−ΔCt^) > 0.01.	It predicts 30-day mortality (AUC: 0.78) better than clinical liver markers. It is an early biomarker of acute liver dysfunction; its levels rise earlier and with greater sensitivity than bilirubin or ALT. It is an independent predictor: HR of 4.4 to 5.4 for mortality in multivariate analysis [231].
**MDA**	Ma et al. [28] In vivo (LPS-induced C57BL/6 mice) and in vitro (MLE-12 epithelial cells).	Homogenized lung tissue and cell lysis; 6, 12, 24 and 48 h post-injury.	Peak at 6 h: ~2.8 µmol/g (LPS) vs. ~1.8 µmol/g (Sham) (*p* < 0.001). Levels gradually decrease at 12–48 h due to compensation.	Acute Phase Marker: Indicates a rapid and intense activation of lipid peroxidation (ferroptosis) within the first 6 h of ARDS, correlating with glutathione (GSH) depletion [28]. It validates the occurrence of lethal oxidative damage in the septic pulmonary epithelium. Its elevation confirms the failure of antioxidant systems (GPX4) and the execution of ferroptosis [237].
**Machine learning models**	Liu et al. [228] *n*: 942 patients (MIMIC-IV). Retrospective. ML: Random Forest (best performance), XGBoost, KNN.	Serum; upon admission to ICU.	ACAG High: >20.8 mmol/L Association: Linear risk of mortality for every 1 mmol/L increase.	The ML model integrates ACAG with scores (SOFA, APS III) to predict 28-day mortality (AUC = 0.73), useful in patients with hypoalbuminemia where the normal Anion Gap fails [228]. Other models predict death at 28 or 90 days with high accuracy (AUC 0.802 in validation), outperforming traditional clinical models (SOFA) [80,234].
**VOCS**	Zhang et al. [232] *n*: 499 (357 derivation, 142 validation). Multicenter observational design. ML: Random Forest for variable selection.	Exhaled air (VOCs); first 48 h of mechanical ventilation.	Decreased concentrations of specific VOCs in ARDS. Performance: AUC of 0.63 in external validation.	Emerging tool. Diagnosis (Limited): Although it distinguishes ARDS from controls, the ML model did not achieve sufficient accuracy for routine clinical use, even when combined with clinical scales (LIPS) [232].
**circRNAs**	Sun et al. [229] *n* = 38 ARDS (severe pneumonia) vs. 38 healthy subjects (Validation). Design: Discovery cohort microarray (*n* = 4) and RT-qPCR validation.	BALF and Plasma Exosomes; <48 h from diagnosis.	The relative expression values (ΔCt) were used to construct the ROC curves and determine the optimal cutoff points: hsa_circRNA_042882: AUC ≈ 0.805, sensitivity 83.5%, specificity 79.9%. hsa_circRNA_104034: AUC ≈ 1.0, with very high sensitivity and specificity.	It distinguishes ARDS from controls with excellent accuracy (especially in BALF). They regulate hypoxia and inflammation pathways (HIF-1 and NF-κB axes) by acting as miRNA sponges [229]. In models of pneumonia-induced sepsis, high levels of Circ-CTD-2281E23.2 predict higher 28-day mortality (AUC = 0.664) and correlate with SOFA and APACHE II scores and inflammatory markers (IL-6, PCT) [235]. In in vitro models, they regulate vascular permeability and the shear stress response through ceRNA networks, affecting angiogenesis and cell adhesion in ARDS [11].
**lncRNAs** **(HOXA-AS2)**	Quan & Gao [79] *n* = 122 sepsis (32 with ARDS, 90 without ARDS) vs. 101 controls. Case–control, RT-qPCR and cell models.	Serum; upon admission (within 24 h).	Downregulation in Sepsis and even lower in ARDS. ARDS diagnosis AUC = 0.843; Mortality: AUC = 0.911.	Dual Prediction: Identifies septic patients at risk of ARDS and death within 28 days (HR = 5.380). Mechanism: Low levels are associated with increased inflammation and degradation of the endothelial glycocalyx [79].

ARDS, acute respiratory distress syndrome; miR, microRNA; circRNA, circular RNA; lncRNA, long non-coding RNA; miR-223, microRNA-223; miR-122, microRNA-122; BALF, bronchoalveolar lavage fluid; VOCs, volatile organic compounds; MDA, malondialdehyde; LPS, lipopolysaccharide; GSH, glutathione; GPX4, glutathione peroxidase 4; ACAG, albumin-corrected anion gap; WGCNA, weighted gene co-expression network analysis; ML, machine learning; SVM, support vector machine; KNN, k-nearest neighbors; ICU, intensive care unit; Associations derive from transcriptomic, metabolomic, epigenetic, oxidative stress, breathomics, or machine learning-based approaches and correlate with ARDS diagnosis, severity, mortality, or ventilator-related outcomes; causality cannot be inferred.

## Data Availability

No new data were created or analyzed in this study.

## Supplementary Materials

The following supporting information can be downloaded at: https://www.mdpi.com/article/10.3390/medsci14010134/s1, Table S1: Cellular immune networks governing pulmonary tolerance and inflammatory amplification in ARDS.

## Abbreviations

The following abbreviations are used in this manuscript:

ACAG	Albumin-corrected anion gap
ACM	Alveolar–Capillary Membrane
AEC1	Alveolar Epithelial Cell Type I (Pneumocyte Type I)
AEC2	Alveolar Epithelial Cell Type II (Pneumocyte Type II)
AFC	Alveolar fluid clearance
AGRN	Agrin
ALI	Acute Lung Injury
AMs	Alveolar Macrophages
Ang-1/2	Angiopoietin-1/2
Ang II	Angiotensin II
APACHE II	Acute physiology and chronic health evaluation II
ARDS	Acute Respiratory Distress Syndrome
AUC	Area under the ROC curve
BALF	Bronchoalveolar lavage fluid
BSG (CD147)	Basigin (CD147)
BTN3A2	Butyrophilin Subfamily 3 Member A2
CC16	Club Cell Protein 16
CCW	Chest Wall Compliance
CPAP	Continuous positive airway pressure
CT	Computed tomography
CXCL8	C-X-C motif chemokine ligand 8
CitH3	Citrullinated histone H3
DAD	Diffuse Alveolar Damage
DAMPs	Damage-Associated Molecular Patterns
DCs	Dendritic cells
DECT	Dual-energy computer tomography
DPPC	Dipalmitoylphosphatidylcholine
EAdi	Diaphragm electrical activity
EC	Endothelial Cell
ECM	Extracellular Matrix
ECMO	Extracorporeal membrane oxygenation
EIT	Electrical impedance tomography
ENAH	Enabled Homolog
ESM-1	Endothelial cell-specific molecule 1 (endocan)
EVLW	Extravascular lung water
F2R	Coagulation Factor II Receptor (Protease-Activated Receptor 1, PAR1)
FRC	Functional Residual Capacity
FiO2	Fraction of inspired oxygen
FoxP3	Forkhead box P3
FXR/RXR	Farnesoid X Receptor/Retinoid X Receptor
GGOs	Ground-Glass Opacities
GM-CSF	Granulocyte macrophage colony-stimulating factor
GPX4	Glutathione peroxidase 4
GSH	Glutathione
HA	Hyaluronic acid
HFNO	High-flow nasal oxygen
HMGB1	High-mobility group box 1 protein
HMWM	High-molecular-weight multimers
HPV	Hypoxic Pulmonary Vasoconstriction
HR	Hazard ratio
HRCT	High-resolution computed tomography
IC	Interstitial Cell
ICU	Intensive care unit
IFN	Interferon
IL	Interleukin
IL-1RA	Interleukin-1 receptor antagonist
ILC2s	Innate Lymphoid Cells Type 2
IPTW	Inverse Probability of Treatment Weighting
IV Col	Type IV collagen
KL-6	Krebs von den Lungen-6 (MUC1)
KNN	K-nearest neighbors
LAIR1	Leukocyte-Associated Immunoglobulin-like Receptor 1
LCA	Latent Class Analysis
LIS	Lung injury score
LN	Laminin
LPS	Lipopolysaccharide
LTBR	Lymphotoxin Beta Receptor
LUS	Lung Ultrasound
MDA	Malondialdehyde
ML	Machine learning
MMP	Matrix metalloproteinase
MPO	Myeloperoxidase
MV	Mechanical ventilation
NETs	Neutrophil extracellular traps
NF-kB	Nuclear Factor Kappa-Light-Chain-Enhancer of Activated B Cells
NLR	Neutrophil-to-lymphocyte ratio
NO	Nitric oxide
NOD1/2	Nucleotide-binding Oligomerization Domain-containing proteins 1 and 2
NUB1	NEDD8 Ultimate Buster 1
OR	Odds ratio
PAI-1	Plasminogen activator inhibitor-1
PAMPs	Pathogen-associated molecular patterns
PEEP	Positive end-expiratory pressure
PI3K	Phosphoinositide 3-kinase
PIIINP	N-terminal propeptide of type III procollagen
PME	Pulmonary Microenvironment
PPAR	Peroxisome Proliferator-Activated Receptor
PRRs	Pattern-recognition receptors
RAAS	Renin–angiotensin–aldosterone system
RALE	Radiographic assessment of lung edema
RAR	Retinoic Acid Receptor
ROS	Reactive oxygen species
SDC-1	Syndecan-1
SOFA	Sequential organ failure assessment
SP-A	Surfactant Protein A
SP-B	Surfactant Protein B
SP-D	Surfactant Protein D
STAT3/6	Signal Transducer and Activator of Transcription 3/6
SVM	Support vector machine
Se	Sensitivity
Sp	Specificity
sRAGE	Soluble receptor for advanced glycation end products
TIMP-1	Tissue inhibitor of metalloproteinases-1
TNF-α	Tumor necrosis factor alpha
TLRs	Toll-like receptors
sTM	Soluble thrombomodulin
TEER	Transendothelial electrical resistance
TREM1	Triggering Receptor Expressed on Myeloid Cells 1
TRMs	Tissue-resident memory T cells
Tregs	Regulatory T Cells
VFD	Ventilator-free days
VILI	Ventilator-Induced Lung Injury
VOCs	Volatile organic compounds
WGCNA	Weighted gene co-expression network analysis
WWOX	WW domain-containing oxidoreductase
circRNA	Circular RNA
lncRNA	Long non-coding RNA
miR	MicroRNA
nRBC	Nucleated red blood cells
sPD-L1	Soluble programmed death ligand 1
sRAGE	Soluble receptor for advanced glycation end-products
sTNFR-1	Soluble tumor necrosis factor receptor 1
suPAR	Soluble urokinase plasminogen activator receptor
vWF	von Willebrand Factor
