# Supplementary material for: Current Insights into Clinical, Molecular, and Therapeutic Approaches to Acute Respiratory Distress Syndrome
Source: Med Sci (Basel). 2026 Mar 13;14(1):134. doi: 10.3390/medsci14010134 (PMC13027457; doi:10.3390/medsci14010134)
Supplement: Supplementary file 1 [file medsci-14-00134-s001.zip › medsci-4173633-supplementary.pdf]

Current Insights into Clinical, Molecular, and Therapeutic Approaches to Acute Respiratory Distress Syndrome

Manuel Gonzalez-Plascencia [1], Margarita L. Martinez-Fierro [1,*], Alfredo Salazar de Santiago [2], Ana G. Castañeda-Miranda [3], José I. Badillo-Almaraz [1] and Idalia Garza-Veloz [1,*]

1 Molecular Medicine Laboratory, Academic Unit of Human Medicine and Health Sciences, Universidad Autónoma de Zacatecas, Carretera Zacatecas-Guadalajara Km.6, Ejido la Escondida, Zacatecas 98160, Mexico; manuelgonzalezcharro@gmail.com (M.G.-P.); jibadillo@hotmail.com (J.I.B.-A.).
2 Unidad Académica de Odontología, Universidad Autónoma de Zacatecas, Zacatecas 98160, Mexico; asalazar@uaz.edu.mx (A.S.d.S.).
3 Laboratorio de Magnetismo Ambiental, Posgrado en Ingeniería para la Innovación Tecnológica, Unidad Académica de Ingeniería Eléctrica, Universidad Autónoma de Zacatecas, Zacatecas 98000, Mexico; agmiranda@uaz.edu.mx (A.G.C.-M.).
* Correspondence: margaritamf@uaz.edu.mx (M.L.M.-F.); idaliagv@uaz.edu.mx (I.G.-V.); Tel.: +52-492-56690 (ext. 2102 or 2103) (M.L.M.-F. & I.G.-V.).

Table S1: Cellular immune networks governing pulmonary tolerance and inflammatory amplification in ARDS.

Cellular lineage / key reference	Primary activators	Activation mechanism	Secreted mediators	Functional consequence	Immune response type
**Axis 1: Pulmonary homeostasis and immune tolerance**					
**Alveolar macrophages (M2-like AMs)** [Yang et al. 2022; Olivares-Martínez et al. 2025]	IL-4, IL-13 (Th2-skewed microenvironment)	STAT6-dependent polarization, tolerogenic phenotype	IL-10, TGF-β, PGE-2	Suppression of excessive immune responses, induction of Tregs, maintenance of alveolar homeostasis	Regulatory innate immunity
**Pulmonary dendritic cells (immature/tolerogenic DCs)** [Ma et al. 2023; Kapur et al. 2017; Cao et al. 2024; Sarma et al. 2025]	Non-inflammatory local signals	Low co-stimulatory molecule expression, IL-10 production	IL-10	Preferential differentiation of naïve T cells into Tregs, inhalational tolerance	Innate–adaptive immune interface
**Regulatory T cells (Tregs, FoxP3⁺)** [Xue et al. 2022; Cao et al. 2024; Kapur et al. 2017; Dial et al. 2017]	IL-10, TGF-β derived from AMs and DCs	FoxP3 induction and stabilization	IL-10, TGF-β	Control of pulmonary inflammation, protection of epithelial and endothelial integrity	Peripheral immune tolerance
**Innate lymphoid cells type 2 (ILC2s)** [Rahimi et al. 2022; Gray et al. 2022]	Mild epithelial stress, IL-33, IL-25	Antigen-independent activation	Amphiregulin, IL-13	Epithelial, endothelial repair, restoration of ACM integrity	Reparative innate immunity
**Tissue-resident memory T cells (TRMs)** [Weiskopf et al. 2020; Li et al. 2024; Kholis et al. 2023; Grant et al. 2021]	Prior cognate antigen exposure	Rapid local reactivation	IFN-γ, cytotoxic granules	Long-term local immune surveillance and rapid protection upon reinfection	Local adaptive immunity
**Circulating monocytes (patrolling phenotype)** [Jiang et al. 2020; Rodero et al. 2015; Mould et al. 2021]	Basal vascular signals	Endothelial surveillance without tissue differentiation	Minimal cytokine release	Immunological monitoring of the alveolar–capillary interface	Innate immune surveillance
**Axis 2: Breakdown of tolerance and inflammatory amplification in ARDS**					
**Alveolar epithelial cells (AECs)** [Guo et al. 2021; Jonassen et al. 2024; Sarma et al. 2025; Wei et al. 2025]	PAMPs, DAMPs, cellular stress	PRR engagement (TLRs), NF-κB and MAPK activation	TNF-α, IL-1β, IL-6, IL-8, CXCL8, GM-CSF, alarmins	Amplification of inflammatory signaling and leukocyte recruitment	Innate immunity

**Alveolar macrophages (M1-like AMs)** [Guo et al. 2021; Grant et al. 2021; Sarma et al. 2025; Malainou et al. 2023]	PAMPs, DAMPs, alarmins	TLR signaling → NF-κB / STAT3 pathways	TNF-α, IL-1β, IL-6, IFN-α/β, IL-12, ROS, NO	Initiation and amplification of innate inflammatory responses, loss of immune tolerance, alveolar-capillary barrier dysfunction.	Pro-inflammatory innate immunity
**Activated pulmonary dendritic cells** [Ma et al. 2023; Young et al. 2023; Sarma et al. 2025; Weiskopf et al. 2020]	HMGB1, DAMPs	PI3K/Akt/mTOR-dependent maturation and migration	IL-12, IL-6, TNF-α Th1: IFN- γ Th2: IL-4, IL-5, IL-13	Polarization of naïve T cells toward effector Th1/Th2 responses	Adaptive immune activation
**Neutrophils** [Potey et al. 2019; Middleton et al. 2020; Guo et al. 2021; Kim et al. 2022; Ramasubramanian et al. 2022]	CXCL8, IL-1β, TNF-α, leukotriene B4	Endothelial adhesion, full activation, and NETosis	NETs (DNA, histones), neutrophil elastase, MPO, ROS	Direct epithelial and endothelial injury, alveolo-capillary barrier disruption	Cytotoxic innate immunity
**Inflammatory monocytes** [Jiang et al. 2020; Sarma et al. 2025; Kyriazopoulou et al. 2021]	Chemokine gradients	Tissue recruitment, differentiation in BMDMs, activation	TNF-α, IL-1β, IL-6, CCL2, CCL7	Amplification of lung inflammation and tissue damage	Innate immunity

AECs: alveolar epithelial cells; AMs: alveolar macrophages; ARDS: acute respiratory distress syndrome; CXCL8 (IL-8): C-X-C motif chemokine ligand 8; DAMPs: damage-associated molecular patterns; DCs: dendritic cells; FoxP3: forkhead box P3; GM-CSF: granulocyte–macrophage colony-stimulating factor; HMGB1: high-mobility group box 1 protein; IFN: interferon; ILC2s: innate lymphoid cells type 2; MPO: myeloperoxidase; NETs: neutrophil extracellular traps; NF-κB: nuclear factor kappa-light-chain-enhancer of activated B cells; NO: nitric oxide; PAMPs: pathogen-associated molecular patterns; PI3K: phosphoinositide 3-kinase; PRRs: pattern-recognition receptors; ROS: reactive oxygen species; STAT3/STAT6: signal transducer and activator of transcription 3/6; TLRs: Toll-like receptors; TRMs: tissue-resident memory T cells; Tregs: regulatory T cells.

Yang, Y.; Ma, Y.; Li, Q.; Ling, Y.; Zhou, Y.; Chu, K.; Xue, L.; Tao, S. STAT6 inhibits ferroptosis and alleviates acute lung injury via regulating P53/SLC7A11 pathway. *Cell Death & Disease* **2022**, *13*, 530, doi:10.1038/s41419-022-04971-x.

Olivares-Martínez, E.; Hernández-Ramírez, D.F.; Núñez-Álvarez, C.A.; Meza-Sánchez, D.E.; Chapa, M.; Méndez-Flores, S.; Priego-Ranero, Á.; Azamar-Llamas, D.; Olvera-Prado, H.; Rivas-Redonda, K.I. Polymerized Type I Collagen Downregulates STAT-1 Phosphorylation Through Engagement with LAIR-1 in Circulating Monocytes, Avoiding Long COVID. *International Journal of Molecular Sciences* **2025**, *26*, 1018.

Ma, A.; Feng, Z.; Li, Y.; Wu, Q.; Xiong, H.; Dong, M.; Cheng, J.; Wang, Z.; Yang, J.; Kang, Y. Ferroptosis-related signature and immune infiltration characterization in acute lung injury/acute respiratory distress syndrome. *Respiratory research* **2023**, *24*, 154.

Kapur, R.; Kim, M.; Aslam, R.; McVey, M.J.; Tabuchi, A.; Luo, A.; Liu, J.; Li, Y.; Shanmugabhavananthan, S.; Speck, E.R.; et al. T regulatory cells and dendritic cells protect against transfusion-related acute lung injury via IL-10. *Blood* **2017**, *129*, 2557–2569, doi:10.1182/blood-2016-12-758185.

Cao, F.; Zhang, L.; Zhao, Z.; Shen, X.; Xiong, J.; Yang, Z.; Gong, B.; Liu, M.; Chen, H.; Xiao, H. TM9SF1 offers utility as an efficient predictor of clinical severity and mortality among acute respiratory distress syndrome patients. *Frontiers in Immunology* **2024**, *15*, 1408406.

Sarma, A.; Christenson, S.A.; Shoshana, B.Z.; Oliveira, A.P.; Neyton, L.P.A.; Mick, E.; Sinha, P.; Wilson, J.G.; Moazed, F.; Leligdowicz, A.; et al. Acute Respiratory Distress Syndrome Molecular Phenotypes Have Distinct Lower Respiratory Tract Transcriptomes. *American Journal of Respiratory and Critical Care Medicine* **2025**, *211*, 2352–2362, doi:10.1164/rccm.202407-1454OC.

Xue, M.; Zhang, X.; Chen, J.; Liu, F.; Xu, J.; Xie, J.; Yang, Y.; Yu, W.; Qiu, H. Mesenchymal Stem Cell - Secreted TGF - β1 Restores Treg/Th17 Skewing Induced by Lipopolysaccharide and Hypoxia Challenge via miR - 155 Suppression. *Stem Cells International* **2022**, *2022*, 5522828.

Dial, C.F.; Tune, M.K.; Doerschuk, C.M.; Mock, J.R. Foxp3+ Regulatory T Cell Expression of Keratinocyte Growth Factor Enhances Lung Epithelial Proliferation. *American Journal of Respiratory Cell and Molecular Biology* **2017**, *57*, 162–173, doi:10.1165/rcmb.2017-0019OC.

Rahimi, R.A.; Cho, J.L.; Jakubzick, C.V.; Khader, S.A.; Lambrecht, B.N.; Lloyd, C.M.; Molofsky, A.B.; Talbot, S.; Bonham, C.A.; Drake, W.P. Advancing lung immunology research: An Official American thoracic society workshop report. *American journal of respiratory cell and molecular biology* **2022**, *67*, e1–18.

Gray, J.I.; Farber, D.L. Tissue-resident immune cells in humans. *Annual review of immunology* **2022**, *40*, 195–220.

Weiskopf, D.; Schmitz, K.S.; Raadsen, M.P.; Grifoni, A.; Okba, N.M.A.; Endeman, H.; van den Akker, J.P.C.; Molenkamp, R.; Koopmans, M.P.G.; van Gorp, E.C.M.; et al. Phenotype and kinetics of SARS-CoV-2–specific T cells in COVID-19 patients with acute respiratory distress syndrome. *Science Immunology* **2020**, *5*, eabd2071, doi:doi:10.1126/sciimmunol.abd2071.

Li, G.; Yan, K.; Zhang, W.; Pan, H.; Guo, P. ARDS and aging: TYMS emerges as a promising biomarker and therapeutic target. *Frontiers in Immunology* **2024**, *15*, 1365206.

Kholis, F.N.; Farhanah, N.; Limantoro, C.; Widyastiti, N.S.; Sobirin, M.A. Increased Levels of IFN-γ, PAI-1, and NT-proBNP are Associated with the Occurrence of Hypoxemia in COVID-19. *The Indonesian Biomedical Journal* **2023**, *15*, 341–350.

Grant, R.A.; Morales-Nebreda, L.; Markov, N.S.; Swaminathan, S.; Querrey, M.; Guzman, E.R.; Abbott, D.A.; Donnelly, H.K.; Donayre, A.; Goldberg, I.A.; et al. Circuits between infected macrophages and T cells in SARS-CoV-2 pneumonia. *Nature* **2021**, *590*, 635–641, doi:10.1038/s41586-020-03148-w.

Jiang, Y.; Rosborough, B.R.; Chen, J.; Das, S.; Kitsios, G.D.; McVerry, B.J.; Mallampalli, R.K.; Lee, J.S.; Ray, A.; Chen, W.; et al. Single cell RNA sequencing identifies an early monocyte gene signature in acute respiratory distress syndrome. *JCI Insight* **2020**, *5*, doi:10.1172/jci.insight.135678.

Rodero, M.P.; Poupel, L.; Loyher, P.-L.; Hamon, P.; Licata, F.; Pessel, C.; Hume, D.A.; Combadiere, C.; Boissonnas, A. Immune surveillance of the lung by migrating tissue monocytes. *Elife* **2015**, *4*, e07847.

Mould, K.J.; Moore, C.M.; McManus, S.A.; McCubbrey, A.L.; McClendon, J.D.; Griesmer, C.L.; Henson, P.M.; Janssen, W.J. Airspace Macrophages and Monocytes Exist in Transcriptionally Distinct Subsets in Healthy Adults. *American Journal of Respiratory and Critical Care Medicine* **2021**, *203*, 946–956, doi:10.1164/rccm.202005-1989OC.

Guo, Q.; Zhao, Y.; Li, J.; Liu, J.; Yang, X.; Guo, X.; Kuang, M.; Xia, H.; Zhang, Z.; Cao, L. Induction of alarmin S100A8/A9 mediates activation of aberrant neutrophils in the pathogenesis of COVID-19. *Cell host & microbe* **2021**, *29*, 222–235. e224.

Jonassen, T.B.; Jørgensen, S.E.; Mitchell, N.H.; Mogensen, T.H.; Berg, R.M.; Ronit, A.; Plovsing, R.R. Alveolar cytokines and interferon autoantibodies in COVID-19 ARDS. *Frontiers in Immunology* **2024**, *15*, 1353012.

Wei, J.; Huang, B.; Hu, K.; Bin, X.; Xiang, S. Identification and validation of potential shared diagnostic markers for sepsis-induced ARDS and cardiomyopathy via WGCNA and machine learning. *Frontiers in Molecular Biosciences* **2025**, *12*, 1665387.

Malainou, C.; Abdin, S.M.; Lachmann, N.; Matt, U.; Herold, S. Alveolar macrophages in tissue homeostasis, inflammation, and infection: evolving concepts of therapeutic targeting. *The Journal of Clinical Investigation* **2023**, *133*.

Young, M.D.; Cancio, T.S.; Thorpe, C.R.; Willis, R.P.; Snook, J.K.; Jordan, B.S.; Demons, S.T.; Salinas, J.; Yang, Z. Circulatory HMGB1 is an early predictive and prognostic biomarker of ARDS and mortality in a swine model of polytrauma. *Frontiers in immunology* **2023**, *14*, 1227751.

Potey, P.M.; Rossi, A.G.; Lucas, C.D.; Dorward, D.A. Neutrophils in the initiation and resolution of acute pulmonary inflammation: understanding biological function and therapeutic potential. *The Journal of pathology* **2019**, *247*, 672–685.

Middleton, E.A.; He, X.-Y.; Denorme, F.; Campbell, R.A.; Ng, D.; Salvatore, S.P.; Mostyka, M.; Baxter-Stoltzfus, A.; Borczuk, A.C.; Loda, M. Neutrophil extracellular traps contribute to immunothrombosis in COVID-19 acute respiratory distress syndrome. *Blood, the journal of the American society of hematology* **2020**, *136*, 1169–1179.

Kim, J.; Baalachandran, R.; Li, Y.; Zhang, C.-O.; Ke, Y.; Karki, P.; Birukov, K.G.; Birukova, A.A. Circulating extracellular histones exacerbate acute lung injury by augmenting pulmonary endothelial dysfunction via TLR4-dependent mechanism. *American Journal of Physiology-Lung Cellular and Molecular Physiology* **2022**, *323*, L223–L239.

Ramasubramanian, B.; Kim, J.; Ke, Y.; Li, Y.; Zhang, C.O.; Promnares, K.; Tanaka, K.A.; Birukov, K.G.; Karki, P.; Birukova, A.A. Mechanisms of pulmonary endothelial permeability and inflammation caused by extracellular histone subunits H3 and H4. *The FASEB Journal* **2022**, *36*, e22470.

Kyriazopoulou, E.; Panagopoulos, P.; Metallidis, S.; Dalekos, G.N.; Poulakou, G.; Gatselis, N.; Karakike, E.; Saridaki, M.; Loli, G.; Stefos, A. An open label trial of anakinra to prevent respiratory failure in COVID-19. *Elife* **2021**, *10*, e66125.
